# Comparative Efficacy and Safety of Sulodexide and Other Extended Anticoagulation Treatments for Prevention of Recurrent Venous Thromboembolism: A Bayesian Network Meta-analysis

**DOI:** 10.1055/s-0040-1709731

**Published:** 2020-04-28

**Authors:** Giuseppe Pompilio, Davide Integlia, Joseph Raffetto, Gualtiero Palareti

**Affiliations:** 1 ISHEO SRL, Rome, Italy; 2 Vascular Surgery Research Laboratories, Division of Vascular and Endovascular Surgery, Brigham and Women's Hospital, Harvard Medical School, Boston, Massachusetts, United States; 3 Fondazione Arianna Anticoagulazione, Bologna, Italy

**Keywords:** sulodexide, major bleeding, unprovoked venous thromboembolism, aspirin, network meta-analysis

## Abstract

**Objective** This network meta-analysis (NMA) assesses the clinical comparative efficacy and safety of sulodexide versus direct-acting oral anticoagulants (DOACs), vitamin K antagonist (VKA), and aspirin in patients with an unprovoked venous thromboembolism (VTE).

**Methods** We conducted a literature search in MEDLINE, Embase, and Cochrane Library using both randomized controlled trials (RCTs) and observational studies. Reduction in recurrent deep venous thrombosis (r-DVT), pulmonary embolism (PE), major bleeding (MB), clinically relevant nonmajor bleeding (CRNMB) were the primary efficacy and safety outcomes. Other secondary end points were also included. We performed a fixed, random effects, and hierarchical models Bayesian NMA for each outcome.

**Results** We identified 18 RCTs and seven observational studies. Random models showed sulodexide is the best treatment compared with DOACs, VKA, and aspirin at reducing the risk of CRNMB, for preventing death from any cause, and VTE/PE/myocardial infarction (MI)/stroke with 0.47, 0.81, and 0.65 probabilities, respectively. In the random model sulodexide was the best treatment for reducing the risk of MB with a 0.50 probability and hierarchical model that confirmed favorable results. Random and hierarchical models showed sulodexide and DOACs to be the best treatments for reducing PE risk. Sulodexide was more effective than aspirin for reducing r-DVT with 0.12 and less of 0.0001 probabilities, respectively.

**Conclusion** Sulodexide is more effective for reducing MB and CRNMB, for preventing deaths from any cause, and from VTE/PE/MI/stroke, than other treatments, for both random and hierarchical models. Sulodexide showed to be more effective than aspirin in reducing the risk of r-DVT and PE. Sulodexide's reduction in bleeding while protecting from recurrent DVT risk makes this therapeutic option an important alternative for extended anticoagulation treatment.

## Introduction

Venous thromboembolism (VTE) is a condition encompassing both deep vein thrombosis (DVT) and pulmonary embolism (PE), which occurs when a thrombus forms in a patient's vein, often in the deep veins of the lower limbs or pelvis. Treatment is usually a short course of heparin followed by a longer course of an anticoagulant treatment, typically either a vitamin K antagonist (VKA) or a direct-acting oral anticoagulant (DOAC). 1 For patients who have had the first episode of VTE, the risk of recurrent event, either DVT and/or PE, persists after the cessation of anticoagulant treatment and is particularly high among patients with an unprovoked VTE. 2 Prolonging anticoagulation appears to protect these patients from recurrence (70–90%), but this carries an increased risk of unpredictable bleeding complications, which based on several risk factors can be as low as 0.8% per year (no risk factors) to as high as 6.5% per year (two or more risk factors). 2 In treating VTE with VKA, DOACs, aspirin, and sulodexide, as from CHEST guidelines, clinicians must balance efficacy at preventing recurrence with risk of causing major bleeding (MB). 2 Extended VKA treatment (warfarin, acenocoumarol) can reduce the risk of VTE recurrence over placebo, but has been shown to increase the bleeding risk. 3 DOACs (dabigatran, rivaroxaban, and apixaban) indirectly showed to be more effective, 4 5 6 having a lower bleeding risk than VKA. 6 The aspirin randomized controlled trials (RCTs) versus placebo highlighted lower efficacy in VTE recurrence and less bleeding risk than VKA. 7 8 Sulodexide, a purified glycosaminoglycan containing 80% heparan sulfate (also called fast-moving heparin) and 20% dermatan sulfate, demonstrated in the Secondary Prevention of Recurrent Deep Vein Thrombosis (SURVET) study versus placebo, 9 the highest reduction of bleeding risk compared with DOACs, VKA, and aspirin, but was not conclusively shown to be as effective as the anticoagulants (DOACs and VKA), in reducing the risk of VTE recurrence. 10 However, sulodexide was more effective than aspirin in reducing VTE recurrence. Therefore, there is a need for an increased understanding and knowledge of the advantages and disadvantages of each of these treatment options. This study, utilizing network meta-analysis (NMA) builds on a systematic review of RCTs and observational studies, to explore the benefits and risks incurred while using anticoagulant or antithrombotic-extended treatments to prevent recurrent VTE.

## Methods

### Search Strategy

The literature search was conducted in Medline, EMBASE, and Cochrane Library in June 2019. The search strategy was developed according to the PICO ( Supplementary Appendix 1, Table 1 ) and proper search string was used ( Supplementary Appendix 1, Table 2 ). Randomized clinical trials and observational studies were investigating VTE treatments (aspirin, sulodexide, VKA, and DOACs) in adult patients affected by unprovoked DVT.

### Selection Criteria

Included studies must meet the following criteria:

- Study design: randomized controlled trial and observational studies that involved VTE patients treated with anticoagulant or antithrombotic drugs after first 3 to 6 months from an acute thrombotic event, to prevent VTE recurrences.
- Patients with proximal DVT (iliofemoral and femoral-popliteal, but not calf vein DVT) or PE after anticoagulant treatment: the initial anticoagulant treatment was low-molecular weight heparin (twice daily, weight adjusted), followed by oral anticoagulant for at least 3 months.
- Intervention: the patients in the intervention group were given sulodexide or other drugs (DOACs, VKA, aspirin); patients in the control group were given placebo or one or more drugs used in the intervention; and the follow-up period was at least 3 months.

Studies meeting the following criteria would be excluded:

- Duplicated articles, experimental studies, and case–control studies.
- Single-arm studies (e.g., where all participants receive the same treatment).
- Patients with persistent pulmonary hypertension after PE, those with solid neoplasm or blood disease, those with antiphospholipid antibody syndrome or antithrombin congenital deficit due to genetic defect or acquired, patients with New York Heart Association class III to IV cardiorespiratory failure, or patients with known hypersensitivity to glycosaminoglycans.

### Study Selection and Data Extraction

Two reviewers independently extracted the following data: first author, year of publication, study design, characteristics of patients, data of interventions, efficacy and safety outcomes, adverse effects, and the quality of included studies. Disagreements were resolved by discussion and consensus. A third reviewer was consulted for the decision on inclusion or exclusion for full text evaluation.

### Assessment of Risk Bias

We utilized the Cochrane Risk of Bias Tool, 11 to evaluate the included RCTs, 3 4 5 6 7 8 9 10 12 13 14 15 16 17 18 19 20 21 22 and the Newcastle–Ottawa scale, 23 to evaluate the observational studies. 24 25 26 27 28 29 30 The quality assessment was performed by two reviewers independently, and any disagreement was resolved via consensus.

### Network Meta-analysis

We initially performed an NMA solely on RCTs, before expanding with additional information from observational studies. Due to limited data and impact on the results from the included observational studies, and that many did not contain comparisons to other interventions, the results are presented as a combination of all the studies unless otherwise noted.

#### Geometry of the Network

For each end point, we described the geometry of the network by drawing network diagrams ( Supplementary Appendix 2 ) showing different treatments and comparing each of the trials and/or observational studies. Separate diagrams were necessary as different studies often reported only selection of the outcomes of interest.

#### End Points of Interest

There were 14 potential end points of interest. Primary outcomes were incidence of recurrent deep venous thrombosis (r-DVT) and PE. Secondary outcomes were distal thrombosis (e.g., calf vein thrombosis); myocardial infarction (MI); stroke; and ischemia. Safety outcomes included MB and clinically relevant nonmajor bleeding (CRNMB); other bleeding leading to drug interruption; death from VTE, MI, or stroke; death from cardiovascular disease (CVD); death from bleeding; death from other causes; and death from any cause. There was not enough data for meaningful analysis for the secondary outcomes of distal thrombosis or ischemia, or for the safety outcome “other bleeding leading to drug interruption”; therefore, these outcomes were not assessed.

#### Pairwise Meta-analysis

Where possible, pairwise meta-analyses were used to explore direct comparisons between: DOAC versus VKA (five studies); DOAC versus placebo (three studies); VKA versus placebo (three studies); and aspirin versus placebo (two studies). There were some suggestions of between-study heterogeneity when carrying out meta-analysis of MB for DOAC versus placebo, but otherwise there was no evidence of any heterogeneity.

#### Network Meta-analysis

For each end point, we performed three NMAs.

1. Fixed-effect NMA of treatment class (i.e., sulodexide vs. DOAC vs. aspirin vs. VKA vs. placebo).
2. Random-effect NMA of treatment class (i.e., sulodexide vs. DOAC vs. aspirin vs. VKA vs. placebo).
3. Hierarchical Bayesian NMA of treatment drug, treating each drug as clustered within its treatment class. Apixaban and rivaroxaban were included in the NMA with each having two different dosages ( Supplementary Appendix 1, Table 3 ). 31 32

All outcomes were binary and logistic regression models were used to analyze the data.

For each model and outcome, we report the following:

- Deviance information criterion (DIC): is a hierarchical modeling generalization of the Akaike information criterion. It is particularly useful in Bayesian model selection problems where the posterior distributions of the models have been obtained by Markov Chain Monte Carlo simulation. 33
- Pairwise odds ratios (OR) for incidence of the outcome.
- the probability that each treatment is “best” P (best).
- the probability that each treatment is “worst” P (worst).
- Surface under the cumulative ranking curve (SUCRA) for each treatment.
- Average rank (AR) for each treatment.

#### Choice of Priors for Each Model

For the binary model, a diffuse prior for trial baselines on the logit scale FO1 and a weakly informative priors for treatment effects FO2 were used. A diffuse prior for the between-trial precision FO3 (the between-trial standard deviation FO4) and a weakly informative prior for the treatments within a specific treatment class FO5 were used for random-effects model and hierarchical model, respectively.

#### Number of Iterations, and Reasons Why

For the binary models, we ran 20,000 iterations, discarding the first 10,000 iterations as burn-in. There were excessive number of iterations, used because estimates of all considered factors had reached convergence (Rhat == 1) and computer time was acceptable (<30 seconds per model).

#### Software

R (version 3.5.2) was used for data processing and preparation, and pairwise meta-analysis. WinBUGs version 14 was used to fit NMA models.

### Assessing Consistency

Consistency models were used to assess the consistency of the evidence base, on the fixed-effect model only. We considered that there was lack of consistency where the DIC of the consistency model was 5 or more less than the DIC of the standard model ( Supplementary Appendix 1, Table 4 ).

There was evidence of inconsistency in the primary outcome of the incidence of DVT. Pairwise (log) ORs for the incidence of DVT from the standard fixed-effects model and from the consistency model were compared. In the consistency model, indirect comparisons were not allowed to affect the results. For example, the OR for the incidence of DVT on sulodexide against that of DOAC was based only on direct comparisons of sulodexide to DOAC, and prior distribution. As there were no direct comparisons of sulodexide to DOAC, or indeed of several other treatment arms, many results from the consistency model were based solely on the diffuse prior information. The consistency model and the standard fixed-effects model gave reasonably precise results for five direct comparisons ( Supplementary Appendix 1, Table 5 ): each active treatment against placebo, plus VKA versus DOAC and aspirin versus DOAC. Comparison against the network diagram shows that these comparisons are the ones where there are trials making them direct comparisons. Direct and indirect evidence appeared broadly in agreement, apart from DOAC versus placebo (where direct evidence suggested DOAC caused larger reductions in DVT recurrence than the indirect evidence) and VKA versus placebo (where direct evidence suggested VKA caused smaller reductions in DVT recurrence than the indirect evidence).

Pragmatically, given that for many pairwise comparisons there are no direct comparisons and that the differences between the consistency and standard models impact the strength rather than direction of associations; it seems reasonable to base further inference on the standard models that assume indirect treatment comparisons are valid.

### Impact of Age and Sex on Outcomes

Logistic regression was used to explore whether age and sex had an impact on outcomes, specifically for r-DVT, PE, MB, and death from any cause. We could not detect differences in safety and efficacy by age or sex, but the available data did not allow this question to be satisfactorily addressed. The only available data were aggregate trial-arm-level information. Factors such as average age, proportion of males, and whether the trial arm was an active treatment (as opposed to placebo) were included as exposures, as was the length-of-follow-up. A random effect was included to allow for clustering of results within specific trials. A model including type of treatment rather than simply active/placebo was considered but did not converge. There were no significant associations between average age and proportion of females and ORs of any of the considered outcomes. Summaries of the logistic regressions used to explore the possible influence of age and sex are presented in Supplementary Appendix 1 (Table 6) .

### Presentation of Results

For each NMA, we estimated pairwise mean differences (continuous end points) or ORs (binary end points) and used these to present results, accompanied with appropriate 95% credible (confidence) intervals. We also presented the probability that certain treatments are best under certain measures. This means that a higher probability indicates that the treatment is likely better at reducing a negative outcome, such as a stroke or an MB. We additionally estimated the SUCRA and average treatment rankings to explore potential orderings of treatment hierarchy. SUCRA is a numerical representation of the overall probability that a drug will occupy the top rank or at least one of the top ranks. The values range from 0 to 100% (higher the SUCRA number more favorable the outcome). Using SUCRA to rank the treatments must be treated with caution as they can arise from evidence that has low certainty. A set of SUCRA rankings can be the same whether they come from high-quality studies or low-quality studies, and the method itself does not differentiate the quality of the study but only the probability of ranking. Therefore, SUCRA results are only as robust as the data they are based on. 34 Average treatment rankings indicate the mean ranking of the treatments across the iterations of the model. The lower the ranking of the numerical value, more favorable is the outcome (e.g., 2.10 being better than 8.80). Combining this with the SUCRA rating provides an indication of the uncertainty in the results; if a treatment has a high SUCRA probability but a low average ranking (higher numerical value) or vice versa, these divergent results may reflect lack of certainty or underlying uncertainty due to having a high probability of being in the top ranks or the bottom ranks. Both random and hierarchical Bayesian models have been reported in the manuscript. Even if random model had a lower DIC (so it is preferable) compared with the hierarchical model, the latter (beyond treating each drug as clustered within its treatment class) allows also to interpret together the RCTs and observational studies modeling between-studies heterogeneity of treatment effects within each study design and across all study designs.

#### Assessing Heterogeneity

Heterogeneity among the comparative studies was assessed using the *I* ^2^ statistic. This statistic indicates what percentage of the observed between-study variability is due to heterogeneity rather than a chance. Heterogeneity in this study was categorized as no heterogeneity when *I* ^2^ = 0 to 25%; moderate heterogeneity when *I* ^2^ = 25 to 50%; large heterogeneity when *I* ^2^ = 50 to 75%; and extreme heterogeneity when *I* ^2^ = 75 to 100%.

## Results

### Systematic Review and Study Characteristics

**Fig. 1:**
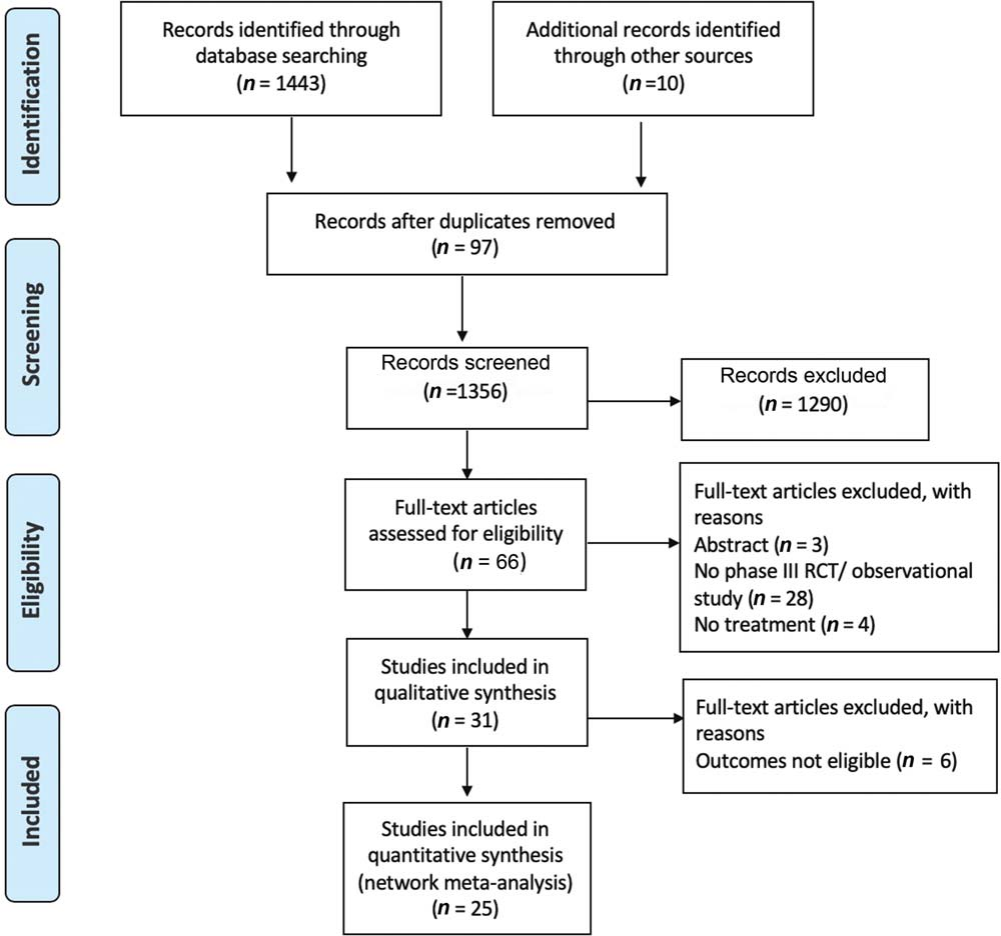
PRISMA diagram. PRISMA, preferred reporting items for systematic reviews and meta-analyses.

A total of 25 studies were included in the NMA ( Fig. 1 ) and the characteristics of which are summarized in Supplementary Appendix 3 (Table 1) .

## Main Results

**Fig. 2:**
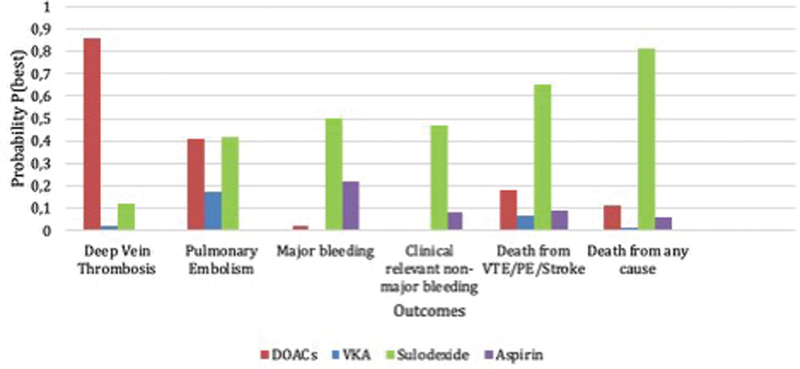
Bar graph showing the probability to be better for each therapeutic approach for the main outcomes.

Considering both random and hierarchical model, the results of NMA showed that sulodexide as a therapeutic approach had a higher probability to be better than those of DOACs, VKA, and aspirin in some of the key outcomes (MB, clinically relevant non-MB, death from VTE/PE/MI/stroke, and death from any cause). The results for random model are shown in the bar graph ( Fig. 2 ) as the probability for each treatment to be the best on specific outcome. These results are also consistent with the probability and SUCRA score statistics in the hierarchical model.

### Recurrent Deep Vein Thrombosis

The network of studies reporting DVT recurrences was well-connected, with many direct and indirect comparisons ( Supplementary Appendix 2, Fig. 1 ). Most were through a single study, and there were no observational studies contributing data. Direct oral anticoagulants appeared to be the most effective drug for reducing DVT ( Table 1 ), with each model giving over 0.86 probability that DOACs were the most effective. VKAs appeared to be second most effective, followed by sulodexide. This order is mirrored in the SUCRA rankings as well. Due to greater uncertainty, given that only one study of sulodexide was included, there were nonzero probabilities that sulodexide was more effective than DOACs and that sulodexide was less effective than placebo. Drug-specific results were similar to the class-based results ( Table 1 ; Supplementary Appendix 4, Table 1 ). In particular, DOACs appeared to have the most effective intervention with the highest SUCRA scores. There was greater spread in the efficacy of VKA treatment, with unspecified VKA regimens appearing less effective than specified drugs. The results of NMA for this outcome are consistent with the scientific literature in which DOACs (dabigatran, rivaroxaban, apixaban, edoxaban) have indirectly been shown to be more effective than VKA for reducing the risk of recurrent VTE. 4 5 6

**Table 1:** Main results for r-DVT, all studies

Statistic	Fixed model estimate (CI 95%)	Random model estimate (CI 95%)	Hierarchical estimate (CI 95%)
DIC	233	223	231
OR sulodexide vs. placebo	0.48 (0.23, 0.96)	0.47 (0.13, 1.88)	0.51 (0.16, 1.72)
OR DOAC vs. placebo	0.23 (0.18, 0.31)	0.22 (0.12, 0.37)	0.19 (0.1, 0.34)
OR VKA vs. placebo	0.33 (0.25, 0.46)	0.34 (0.2, 0.58)	0.36 (0.18, 0.67)
OR aspirin vs. placebo	0.74 (0.53, 1.04)	0.7 (0.34, 1.35)	0.71 (0.35, 1.28)
OR DOAC vs. sulodexide	0.48 (0.23, 1.1)	0.47 (0.1, 1.81)	0.38 (0.1, 1.47)
OR VKA vs. sulodexide	0.69 (0.33, 1.64)	0.73 (0.18, 3.02)	0.66 (0.18, 2.8)
OR aspirin vs. sulodexide	1.53 (0.68, 3.53)	1.5 (0.33, 5.99)	1.31 (0.35, 4.92)
OR VKA vs. DOAC	1.44 (1.14, 1.77)	1.57 (1, 2.59)	1.83 (0.94, 3.89)
OR aspirin vs. DOAC	3.2 (2.09, 4.91)	3.26 (1.54, 7.24)	3.59 (1.81, 7.64)
OR aspirin vs. VKA	2.22 (1.44, 3.43)	2.05 (0.85, 4.65)	1.97 (0.83, 4.49)
P (placebo best)	<0.0001	<0.0001	<0.0001
P (sulodexide best)	0.04	0.12	0.06
P (DOAC best)	0.96	0.86	0.9
P (VKA best)	<0.0001	0.02	0.03
P (aspirin best)	<0.0001	<0.0001	<0.0001
P (placebo worst)	0.94	0.76	0.75
P (sulodexide worst)	0.02	0.1	0.14
P (DOAC worst)	<0.0001	<0.0001	<0.0001
P (VKA worst)	<0.0001	<0.0001	<0.0001
P (aspirin worst)	0.04	0.14	0.1
SUCRA (placebo)	0.02	0.06	<0.0001
SUCRA (sulodexide)	0.51	0.51	0.17
SUCRA (DOAC)	0.99	0.96	0.95
SUCRA (VKA)	0.7	0.67	0.36
SUCRA (aspirin)	0.28	0.29	0.02
Average rank (placebo)	4.94	4.74	4.73
Average rank (sulodexide)	2.94	2.95	3.14
Average rank (DOAC)	1.04	1.15	1.1
Average rank (VKA)	2.19	2.32	2.31
Average rank (aspirin)	3.89	3.84	3.71

Abbreviations: CI, credible interval; DIC, deviance information criterion; DOAC, direct-acting oral anticoagulant; OR, odds ratio; P, probability; r-DVT, recurrent-deep venous thrombosis; SUCRA, surface under the cumulative ranking curve; VKA, vitamin K antagonist.

### Pulmonary Embolism

The network of studies reporting PE was well-connected, with many direct and indirect comparisons. Most were through a single study, and there were no observational studies contributing data ( Supplementary Appendix 2, Fig. 2 ). DOACs, VKA, and sulodexide were all potentially the best treatment for reducing PE risk ( Table 2 ), with DOACs having the highest probability in both random and hierarchical model, followed by sulodexide, VKA, and aspirin. The treatments with the highest SUCRA score in the random model were DOACs, followed by VKA and sulodexide with similar score to VKA, but sulodexide showed also the highest probability to be the best in SUCRA score than VKA in hierarchical model. These results are aligned with the European Society of Cardiology guidelines 2019 (ESC). 35 As with the r-DVT results, drug-specific results and class-based results were similar ( Table 2 ; Supplementary Appendix 4, Table 2 ). In particular, most DOACs appeared to have similar effectiveness. There was more spread in the efficacy of VKA treatments.

**Table 2:** Main results for PE, all studies

Statistic	Fixed model estimate (CI 95%)	Random model estimate (CI 95%)	Hierarchical estimate (CI 95%)
DIC	195	195	203
OR sulodexide vs. placebo	0.51 (0.11, 1.8)	0.46 (0.08, 1.72)	0.43 (0.11, 2.24)
OR DOAC vs. placebo	0.41 (0.28, 0.58)	0.4 (0.25, 0.59)	0.33 (0.18, 0.56)
OR VKA vs. placebo	0.43 (0.3, 0.62)	0.43 (0.27, 0.64)	0.41 (0.22, 0.75)
OR aspirin vs. placebo	0.75 (0.49, 1.16)	0.76 (0.47, 1.21)	0.8 (0.44, 1.7)
OR DOAC vs. sulodexide	0.83 (0.21, 3.91)	0.87 (0.21, 4.8)	0.76 (0.14, 3.22)
OR VKA vs. sulodexide	0.89 (0.22, 4.15)	0.93 (0.24, 5.06)	0.94 (0.17, 4.21)
OR aspirin vs. sulodexide	1.5 (0.36, 7.23)	1.7 (0.38, 10.33)	1.92 (0.36, 8.09)
OR VKA vs. DOAC	1.07 (0.86, 1.32)	1.08 (0.81, 1.48)	1.26 (0.69, 2.38)
OR aspirin vs. DOAC	1.82 (1.15, 3.1)	1.94 (1.13, 3.45)	2.45 (1.27, 5.82)
OR aspirin vs. VKA	1.72 (1.05, 2.89)	1.77 (1.03, 3.21)	1.95 (0.89, 5.04)
P (placebo best)	<0.0001	<0.0001	<0.0001
P (sulodexide best)	0.38	0.42	0.33
P (DOAC best)	0.42	0.41	0.53
P (VKA best)	0.2	0.17	0.14
P (aspirin best)	<0.0001	<0.0001	<0.0001
P (placebo worst)	0.76	0.72	0.65
P (sulodexide worst)	0.15	0.15	0.12
P (DOAC worst)	<0.0001	<0.0001	<0.0001
P (VKA worst)	<0.0001	<0.0001	<0.0001
P (aspirin worst)	0.09	0.12	0.23
SUCRA (placebo)	0.07	0.07	<0.0001
SUCRA (sulodexide)	0.59	0.62	0.4
SUCRA (DOAC)	0.83	0.82	0.72
SUCRA (VKA)	0.71	0.7	0.37
SUCRA (aspirin)	0.3	0.29	0.01
Average rank (placebo)	4.74	4.7	4.62
Average rank (sulodexide)	2.64	2.52	2.54
Average rank (DOAC)	1.69	1.71	1.57
Average rank (VKA)	2.16	2.21	2.3
Average rank (aspirin)	3.78	3.85	3.97

Abbreviations: CI, credible interval; DIC, deviance information criterion; DOAC, direct-acting oral anticoagulant; OR, odds ratio; P, probability; PE, pulmonary embolism; SUCRA, surface under the cumulative ranking curve; VKA, vitamin K antagonist.

### Distal Thrombosis (Calf Veins) or Superficial Venous Thrombosis

An NMA of distal thrombosis or superficial venous thrombosis (SVT) was not possible. Three studies tracked distal or SVT events ( Supplementary Appendix 2, Fig. 3 ), but the number of events were so low that the results were almost entirely determined by the priors used in the meta-analysis.

### Myocardial Infarction

Myocardial infarctions were reported by seven RCTs ( Supplementary Appendix 2, Fig. 4 ).VKAs appeared to be the most effective drug for reducing MI ( Supplementary Appendix 4, Table 3 ), with each model giving at least a 0.52 probability that VKA was most effective. Sulodexide appeared to be second most effective, followed by DOACs. The SUCRA scores followed the same order of treatments. Drug-specific results were similar to the class-based results ( Supplementary Appendix 4, Tables 3 and 8 ). The VKA appeared to be the most effective wherein all of the analyses had the highest SUCRA scores, while sulodexide and DOACs had similar but lower SUCRA scores. There was more spread in the potential efficacy of DOACs with SUCRA scores ranging from 0.31 to 0.56.

### Stroke

Strokes were reported by eight RCTs ( Supplementary Appendix 2, Fig. 5 ).

The results for the prevention of strokes were similar to those for DVT, where DOACs had by far the highest SUCRA scores and appeared to be the most effective drug for reducing strokes ( Supplementary Appendix 4, Table 5 ). With regard to the probability of DOACs being the most effective, two models indicated at least a 0.60 probability and the other 0.47, which shows that treatments with DOACs were the most effective. Aspirin appeared to be second most effective, followed by VKA, though the difference was minimal in both the SUCRA scores with probabilities of being the most effective. Sulodexide appeared to be the least effective; however, due to the limited data for sulodexide there was also a nonzero probability that it was the most effective. Drug-specific results were similar to the class-based results ( Supplementary Appendix 4, Tables 5 and 6 ), with DOACs and aspirin appearing to be the most effective.

### Acute Ischemia

An NMA of acute ischemia of the lower limbs was not possible. Two RCTs tracked distal or SVT events ( Supplementary Appendix 2, Fig. 6 ), but the number of events were so low that the results were almost entirely determined by the priors used in the meta-analysis.

### Major Bleeding

The network of studies reporting MB was well-connected, with many direct and indirect comparisons. Most were through a single study, and there was one observational study contributing data ( Supplementary Appendix 2, Fig. 7 ). Aspirin did not appear to increase the risk of MB compared with placebo ( Table 3 ). Sulodexide showed the highest probability to be the best treatment when compared with aspirin, DOACs, and VKA, but it had a lower SUCRA and AR compared with aspirin. Because the results of random model were divergent, due to only one study for sulodexide, both hierarchical model for each class and specific drugs ( Supplementary Appendix 4, Tables 3 and 7 ) should be considered to assess sulodexide performance. Since SUCRA and AR showed the highest and lowest values for sulodexide for both hierarchical models, respectively, this indicates that sulodexide is more effective than aspirin in hierarchical model to reduce the risk of MB. DOACs and VKA both appeared to raise the risk of MB, with VKA raising the greatest risk. As may have been expected, VKA seemed most likely to be the treatment with greatest risk of increasing the MB.

**Table 3:** Main results for major bleeding, all studies

Statistic	Fixed model estimate (CI 95%)	Random model estimate (CI 95%)	Hierarchical estimate (CI 95%)
DIC	169	166	185
OR sulodexide vs. placebo	0.95 (0.001, 303.349)	0.87 (0, 664.44)	0.2 (0, 10.48)
OR DOAC vs. placebo	1.6 (0.84, 2.89)	1.59 (0.81, 3.3)	1.51 (0.72, 3.44)
OR VKA vs. placebo	2.71 (1.47, 4.77)	2.75 (1.46, 5.44)	2.82 (1.31, 6.26)
OR aspirin vs. placebo	1.1 (0.45, 2.36)	1.09 (0.38, 3.01)	1.03 (0.43, 2.59)
OR DOAC vs. sulodexide	1.64 (0.01, 866.08)	1.76 (0. 1025.49)	7.87 (0.14, 558.82)
OR VKA vs. sulodexide	2.8 (0.01, 1616.03)	3.12 (0, 1541.37)	13.9 (0.26, 1001.92)
OR aspirin vs. sulodexide	1.14 (0, 827.86)	1.23 (0, 559.34)	5.62 (0.1, 341.48)
OR VKA vs. DOAC	1.69 (1.36, 2.12	1.73 (1.18, 2.51)	1.85 (0.93, 3.55)
OR aspirin vs. DOAC	0.7 (0.28, 1.68)	0.68 (0.23, 1.91)	0.69 (0.24, 1.78)
OR aspirin vs. VKA	0.41 (0.16, 0.99)	0.39 (0.13, 1.16)	0.38 (0.12, 1.08)
P (placebo best)	0.28	0.26	0.13
P (sulodexide best)	0.49	0.5	0.72
P (DOAC best)	0.02	0.02	0.02
P (VKA best)	<0.0001	<0.0001	<0.0001
P (aspirin best)	0.22	0.22	0.12
P (placebo worst)	<0.0001	<0.0001	<0.0001
P (sulodexide worst)	0.38	0.36	0.16
P (DOAC worst)	<0.0001	<0.0001	0.03
P (VKA worst)	0.6	0.61	0.8
P (aspirin worst)	0.01	0.02	0.02
SUCRA (placebo)	0.75	0.74	0.36
SUCRA (sulodexide)	0.55	0.57	0.73
SUCRA (DOAC)	0.43	0.43	0.08
SUCRA (VKA)	0.1	0.1	<0.0001
SUCRA (aspirin)	0.67	0.66	0.33
Average rank (placebo)	1.99	2.03	2.35
Average rank (sulodexide)	2.8	2.73	1.9
Average rank (DOAC)	3.28	3.27	3.44
Average rank (VKA)	4.59	4.59	4.77
Average rank (aspirin)	2.33	2.38	2.54

Abbreviations: CI, credible interval; DIC, deviance information criterion; DOAC, direct-acting oral anticoagulant; OR, odds ratio; P, probability; SUCRA, surface under the cumulative ranking curve; VKA, vitamin K antagonist.

Drug-specific results were similar to the class-based results ( Supplementary Appendix 4, Tables 3 and 7 ) with placebo and sulodexide having the highest SUCRA scores.

### Clinically Relevant Nonmajor Bleeding

The network of studies reporting CRNMB included 13 RCTs, with many direct and indirect comparisons. Most were through a single study, and there were no observational studies contributing data ( Supplementary Appendix 2, Fig. 8 ). Sulodexide did not appear to increase risk of CRNMB compared with placebo ( Table 4 ). There was minimal evidence of risks associated with aspirin, and it may either raise, reduce, or not change the risk of CRNMB (with nonzero probability of being either the best or worst treatment). Similar to MB, DOACs and VKA both appeared to raise the risk of CRNMB, with VKA raising the risk more. VKA seemed most likely to be the worst treatment for the risk of bleeding. Drug-specific results were similar to the class-based results ( Supplementary Appendix 4, Tables 4 and 8 ) with sulodexide and placebo having the highest SUCRA scores among the treatments.

**Table 4:** Main results for clinically relevant nonmajor bleeding, all studies

Statistic	Fixed model estimate (CI 95%)	Random model estimate (CI 95%)	Hierarchical estimate (CI 95%)
DIC	209	180	192
OR sulodexide vs. placebo	0.95 (0.13, 7.63)	1.08 (0.12, 10.3)	0.59 (0.02, 3.61)
OR DOAC vs. placebo	2.33 (1.7, 3.34)	2.31 (1.32, 4.05)	2.31 (1.4, 4.05)
OR VKA vs. placebo	3.05 (2.2, 4.45)	3.02 (1.51, 5.81)	3.1 (1.5, 6.41)
OR aspirin vs. placebo	1.55 (0.86, 2.73)	1.57 (0.71, 3.78)	1.9 (0.88, 4.03)
OR DOAC vs. sulodexide	2.45 (0.29, 19.56)	2.1 (0.22, 21.03)	3.91 (0.58, 119.76)
OR VKA vs. sulodexide	3.17 (0.39, 25.23)	2.77 (0.29, 26.6)	5.48 (0.71,162.18)
OR aspirin vs. sulodexide	1.57 (0.19, 13.01)	1.52 (0.14, 14.24)	3.2 (0.36, 92.93)
OR VKA vs. DOAC	1.31 (1.19, 1.44)	1.32 (0.89, 1.93)	1.34 (0.72, 2.45)
OR aspirin vs. DOAC	0.66 (0.4, 1.1)	0.67 (0.31, 1.55)	0.81 (0.39, 1.77)
OR aspirin vs. VKA	0.51 (0.3, 0.85)	0.5 (0.21, 1.31)	0.61 (0.25, 1.57)
P (placebo best)	0.45	0.45	0.31
P (sulodexide best)	0.51	0.47	0.67
P (DOAC best)	<0.0001	<0.0001	<0.0001
P (VKA best)	<0.0001	<0.0001	<0.0001
P (aspirin best)	0.03	0.08	0.02
P (placebo worst)	<0.0001	<0.0001	<0.0001
P (sulodexide worst)	0.14	0.18	0.05
P (DOAC worst)	<0.0001	0.04	0.1
P (VKA worst)	0.85	0.73	0.74
P (aspirin worst)	<0.0001	0.05	0.1
SUCRA (placebo)	0.85	0.85	0.63
SUCRA (sulodexide)	0.71	0.66	0.75
SUCRA (DOAC)	0.31	0.34	0.02
SUCRA (VKA)	0.04	0.08	<0.0001
SUCRA (aspirin)	0.58	0.57	0.09
Average rank (placebo)	1.58	1.62	1.73
Average rank (sulodexide)	2.16	2.35	1.65
Average rank (DOAC)	3.74	3.65	3.74
Average rank (VKA)	4.85	4.67	4.67
Average rank (aspirin)	2.66	2.72	3.2

Abbreviations: CI, credible interval; DIC, deviance information criterion; DOAC, direct-acting oral anticoagulant; OR, odds ratio; P, probability; SUCRA, surface under the cumulative ranking curve; VKA, vitamin K antagonist.

### Death from VTE/PE/MI/Stroke

The network of studies reporting death from VTE, PE, MI, or stroke was well-connected and included 17 RCTs with many direct and indirect comparisons. Most were through a single study, and there were no observational studies contributing data ( Supplementary Appendix 2, Fig. 9 ).When it came to preventing death from VTE, PE, MI, or stroke, sulodexide appeared to be the most effective drug with all models suggesting there was at least a 0.65 probability of this being the case ( Table 5 ), and also these models supported sulodexide having the highest SUCRA score. DOACs appeared to be the second most effective with either VKA or aspirin being the third most effective. There was also a nonzero probability that sulodexide was the least effective though, due to the uncertainty stemming from only one study being in the meta-analysis. Drug-specific results were similar to the class-based results ( Supplementary Appendix 4, Tables 5 and 9 ) with sulodexide again having the highest SUCRA score (0.77).

**Table 5:** Main results for death from VTE/PE/MI/stroke, all studies

Statistic	Fixed model estimate (CI 95%)	Random model estimate (CI 95%)	Hierarchical estimate (CI 95%)
DIC	150	150	157
OR sulodexide vs. placebo	0.32 (0.03, 2.2)	0.38 (0.03, 3.36)	0.27 (0.02, 2.15)
OR DOAC vs. placebo	0.69 (0.32, 1.32)	0.65 (0.28, 1.42)	0.55 (0.21, 1.39)
OR VKA vs. placebo	0.77 (0.38, 1.51)	0.76 (0.36, 1.66)	0.73 (0.29, 2.02)
OR aspirin vs. placebo	1.01 (0.31, 3.15)	0.96 (0.29, 3.29)	1.47 (0.36, 5.36)
OR DOAC vs. sulodexide	2.13 (0.27, 32)	1.86 (0.15, 26.25)	2.02 (0.22, 24.65)
OR VKA vs. sulodexide	2.39 (0.31, 33.04)	2.11 (0.2, 28.53)	2.66 (0.32, 33.49)
OR aspirin vs. sulodexide	3.19 (0.31, 45.75)	2.67 (0.19, 54.42)	5.08 (0.54, 78)
OR VKA vs. DOAC	1.13 (0.73, 1.74)	1.15 (0.69, 2.15)	1.33 (0.59, 3.69)
OR aspirin vs. DOAC	1.5 (0.42, 5.05)	1.43 (0.4, 5.24)	2.72 (0.54, 10.51)
OR aspirin vs. VKA	1.34 (0.35, 4.49)	1.23 (0.34, 4.93)	2 (0.36, 8.81)
P (placebo best)	<0.0001	0.01	0.01
P (sulodexide best)	0.71	0.65	0.69
P (DOAC best)	0.15	0.18	0.24
P (VKA best)	0.06	0.07	0.05
P (aspirin best)	0.07	0.09	0.01
P (placebo worst)	0.34	0.33	0.22
P (sulodexide worst)	0.1	0.12	0.05
P (DOAC worst)	0.03	0.04	0.01
P (VKA worst)	0.09	0.11	0.09
P (aspirin worst)	0.44	0.4	0.63
SUCRA (placebo)	0.25	0.25	0.05
SUCRA (sulodexide)	0.81	0.77	0.74
SUCRA (DOAC)	0.64	0.64	0.47
SUCRA (VKA)	0.48	0.49	0.19
SUCRA (aspirin)	0.32	0.35	0.05
Average rank (placebo)	4	3.98	3.83
Average rank (sulodexide)	1.75	1.94	1.68
Average rank (DOAC)	2.45	2.44	2.15
Average rank (VKA)	3.07	3.04	3
Average rank (aspirin)	3.74	3.61	4.34

Abbreviations: CI, credible interval; DIC, deviance information criterion; DOAC, direct-acting oral anticoagulant; MI, myocardial infarction; OR, odds ratio; P, probability; PE, pulmonary embolism; SUCRA, surface under the cumulative ranking curve; VKA, vitamin K antagonist; VTE, venous thromboembolism.

### Death from CVD

The network of studies reporting death from CVD included 12 RCTs with both direct and indirect comparisons. Most were through a single study, and there were no observational studies contributing data ( Supplementary Appendix 2, Fig. 10 ). Aspirin appeared to be the most effective drug for reducing deaths from CVD ( Supplementary Appendix 4, Table 10 ), with each model giving over a 0.45 probability that aspirin was the most effective. DOACs and sulodexide appeared to be the second most effective, with two models showing sulodexide to be more effective and the other favoring DOACs. The SUCRA results were similar, however, with this measure favored DOACs over sulodexide as the second most effective treatment. Due to greater uncertainty, sulodexide also had the highest probability of being the least effective treatment. Drug-specific results were similar to the class-based results ( Supplementary Appendix 4, Tables 10 and 11 ) with aspirin having the highest score (0.77). DOACs had higher SUCRA scores than other treatments, apart from aspirin, with apixaban nearly scoring as high (0.72) as aspirin.

### Death from Bleeding

The network of studies reporting death from bleeding included 14 RCTs with both direct and indirect comparisons. Most were through a single study, and there were no observational studies contributing data ( Supplementary Appendix 2, Fig. 11 ). Due to the low number of patients experiencing this outcome, there was a great deal of uncertainty in the results. Aspirin appears to be the most effective treatment in preventing death from bleeding with at least 0.39 probability, but sulodexide and DOACs both have at least 0.24 probability of being the most effective as well ( Supplementary Appendix 4, Table 12 ). According to the SUCRA scores, aspirin and DOACs are most likely to be the most effective.

Drug-specific results were similar to the class-based results ( Supplementary Appendix 4, Tables 12 and 13 ), except that sulodexide had a higher likelihood of being more effective than DOACs.

### Death from Any Cause (Unspecified)

The network of studies reporting death from any cause included 16 RCTs with both direct and indirect comparisons. Most were through a single study, and there was only one observational study contributing data ( Supplementary Appendix 2, Fig. 12 ). Sulodexide again appeared to be the most effective drug with the highest SUCRA score ( Table 6 ), with each model giving at least a 0.69 probability that sulodexide was most effective at reducing death from any cause. The treatment that appeared to be the second most effective was that of DOACs. Sulodexide also had a nonzero probability of being the least effective treatment. Drug-specific results were similar to the class-based results ( Supplementary Appendix 4, Tables 6 and 14 ) with sulodexide having a 0.53 probability of being the most effective.

**Table 6:** Main results for death from any cause (unspecified), all studies

Statistic	Fixed model estimate (CI 95%)	Random model estimate (CI 95%)	Hierarchical estimate (CI 95%)
DIC	176	176	199
OR sulodexide vs. placebo	0.35 (0.06, 1.64)	0.34 (0.05, 1.28)	0.34 (0.04, 1.84)
OR DOAC vs. placebo	0.76 (0.51, 1.18)	0.74 (0.47, 1.19)	0.54 (0.25, 1)
OR VKA vs. placebo	0.87 (0.58, 1.35)	0.86 (0.57, 1.38)	0.81 (0.42, 1.51)
OR aspirin vs. placebo	0.86 (0.53, 1.45)	0.85 (0.49, 1.54)	0.94 (0.42, 2.09)
OR DOAC vs. sulodexide	2.27 (0.45, 14.89)	2.24 (0.52, 15.59)	1.62 (0.25, 13.92)
OR VKA vs. sulodexide	2.56 (0.51, 16.87)	2.63 (0.59, 17.97)	2.49 (0.37, 21.06)
OR aspirin vs. sulodexide	2.5 (0.52, 14.61)	2.56 (0.61, 17.35)	2.73 (0.54, 22.51)
OR VKA vs. DOAC	1.14 (0.93, 1.42)	1.16 (0.91, 1.55)	1.49 (0.75, 3.39)
OR aspirin vs. DOAC	1.12 (0.62, 2.03)	1.14 (0.6, 2.27)	1.78 (0.74, 4.32)
OR aspirin vs. VKA	0.98 (0.55, 1.83)	0.97 (0.52, 1.93)	1.15 (0.46, 2.92)
P (placebo best)	0.01	<0.0001	<0.0001
P (sulodexide best)	0.8	0.81	0.69
P (DOAC best)	0.12	0.11	0.27
P (VKA best)	0.01	0.01	0.03
P (aspirin best)	0.06	0.06	0.01
P (placebo worst)	0.49	0.51	0.42
P (sulodexide worst)	0.08	0.05	0.05
P (DOAC worst)	0.02	0.02	0.01
P (VKA worst)	0.19	0.19	0.15
P (aspirin worst)	0.21	0.23	0.37
SUCRA (placebo)	0.19	0.18	0.01
SUCRA (sulodexide)	0.87	0.89	0.77
SUCRA (DOAC)	0.64	0.65	0.55
SUCRA (VKA)	0.36	0.36	0.12
SUCRA (aspirin)	0.44	0.43	0.06
Average rank (placebo)	4.23	4.29	4.21
Average rank (sulodexide)	1.54	1.44	1.61
Average rank (DOAC)	2.44	2.41	1.94
Average rank (VKA)	3.56	3.57	3.33
Average rank (aspirin)	3.23	3.29	3.92

Abbreviations: CI, credible interval; DIC, deviance information criterion; DOAC, direct-acting oral anticoagulant; OR, odds ratio; P, probability; SUCRA, surface under the cumulative ranking curve; VKA, vitamin K antagonist.

### Heterogeneity

The heterogeneity explored by forest plots can be found in Supplementary Appendix 5 (Fig. 1) . There was no evidence of significant heterogeneity when exploring the number of PE events, and death from bleeding or deaths from all causes for any of the comparisons. There was some moderate heterogeneity for the number of DVT events for the VKA versus DOAC ( *I* ^2^ = 44.56%) and aspirin versus placebo ( *I* ^2^ = 48.35%). For the other two outcomes, number of MB and deaths from VTE events, there was a large heterogeneity for only one comparison in each outcome; DOAC versus placebo for the former ( *I* ^2^ = 59.84%) and VKA versus placebo for the latter ( *I* ^2^ = 53.24%). The heterogeneity was explored by forest plot.

### Publication Bias

To ascertain publication bias, pairwise funnel plots were developed for the number of VTE events, PE events, MB, deaths from VTE events, death from bleeding events, and all cause outcomes ( Supplementary Appendix 5, Fig. 2 ). It is important to note that while the results do not reveal any publication bias for these outcomes, it is generally held that at least five studies are required to detect funnel plot asymmetry. 36 Egger's test was not conducted as its power to detect bias is extremely low with less than five studies.

### Assessment of Risk of Bias

The results of the risk of bias assessments both for the RCTs and the observational studies are presented in Supplementary Appendix 5 (Figs. 3 and 4) . While many of the RCTs were constructed appropriately to minimize bias, five studies did have aspects that introduced bias into the results. 4 5 14 16 20 The bias in these studies can be mainly attributed to the performance bias due to the lack of blinding of study participants, and outcome assessors and selection bias due to the open-label nature of the study.

The bias introduced in the observational studies was mainly attributed to the lack of comparability of the cohorts involved. For six of the studies, 24 25 26 27 28 30 there was no attempt to improve the comparability of the cohorts as the only differentiation used was event outcomes.

### Limitations

Limitations to this study include potential bias introduced from the performance and selection bias in four of the included RCTs and from the failure to ensure comparability between cohorts in six of the observational studies. In addition, since the clustering of drugs belonging to DOACs class was necessary to perform the hierarchical Bayesian analysis an assumption was made, supported by evidence-based literature, 4 5 6 about the similar efficacy of drugs included in DOACs class in which also two different dosages for both apixaban and rivaroxaban were considered. 31 32

## Discussion

The present NMA confirmed that extended therapy with sulodexide reduces the risk of MB more than aspirin as highlighted by hierarchical model. In addition, sulodexide reduces clinically relevant non-MB more than aspirin, DOACs, and VKA in both random and hierarchical model, as recent NMA reported, 10 and sulodexide had a lower risk of death from any cause and from VTE/PE/MI/stroke. Although the analysis showed that DOACs were the best treatment for reducing recurrent DVT episodes and PE, sulodexide reported for the latter end point values very close to those of VKA in the random model, and even probability to be the best treatment and higher SUCRA than VKA in hierarchical model. This result is completely aligned with the 2019 ESC guidelines which recommend sulodexide for extended VTE prophylaxis “in patients who refuse to take or are unable to tolerate any form of oral anticoagulants.” 34 Even if the results supported the use of DOACs, VKA, and aspirin for appropriate treatment strategies to prevent VTE recurrence, just like recent guidelines recommended, 2 sulodexide is shown to be a more effective treatment than aspirin. However, these results should be interpreted with caution in the absence of head-to-head clinical trials comparing sulodexide with other anticoagulation or antithrombotic regimens. Of interest was the finding that sulodexide decreased bleeding events and mortality, as well as having adverse events comparable to placebo, as reported by the SURVET study. One clinical trial and other studies have demonstrated that the bleeding safety of sulodexide, and its reduction of death may be related to antithrombotic, endothelial protection, and anti-inflammatory effects. 9 37 38 39 Sulodexide's antithrombotic effect appears through enhancing the antiprotease activities of both antithrombin (AT III) and heparin cofactor II, with subsequent inhibition of thrombin production. Sulodexide also has endothelial anti-inflammatory effect and a profibrinolytic activity by promoting the release of tissue plasminogen activator, and reducing the activity of plasminogen activator inhibitor, leading to the reduction in circulating fibrinogen. The endothelial protective effect of sulodexide seems to be an important mechanism in preventing VTE because the disruption of the integrity of the endothelium is one of the causes that can lead to the development of VTE. 39

Further research is needed in determining the mechanisms of sulodexide's ability to reduce mortality in patients affected by VTE, with significant and important clinical, patient benefit and wellbeing, economic, and scientific implications.

## Conclusion

This NMA demonstrated that sulodexide is more effective for reducing MB and CRNMB risk, for preventing deaths from any cause and from VTE/PE/MI/stroke, when compared with DOACs, VKA, and aspirin. Results were consistent for both the random and hierarchical models. For MB, the probability of sulodexide to be the best treatment was supported by SUCRA and AR only in the hierarchical model. When considering the prevention of VTE recurrence, DOACs were associated with the highest probabilities of efficacy over VKA, sulodexide, and aspirin, respectively. Sulodexide showed to be more effective than aspirin in reducing the risk of DVT and PE recurrence, reporting very similar values to those of VKA for reduction of PE risk.
