# Supplementary material for: Comparative Efficacy and Safety of Sulodexide and Other Extended Anticoagulation Treatments for Prevention of Recurrent Venous Thromboembolism: A Bayesian Network Meta-analysis
Source: TH Open. 2020 Apr 28;4(2):e80–93. doi: 10.1055/s-0040-1709731 (PMC7188513; doi:10.1055/s-0040-1709731)
Supplement: Supplementary file 1 — Supplementary Material [file 10-1055-s-0040-1709731-s200002.pdf]

# Supplementary Appendix 1: PICO statement

**Table 1** PICO statement

| | |
|---|---|
| Problem (or population) | Population affected by unprovoked deep vein thrombosis (DVT) |
| Intervention | Sulodexide |
| Comparison or control | Placebo or other drugs DOACs (dabigatran, rivaroxaban, apixaban, ximelagatran), VKA (warfarin, acenocoumarol), aspirin |
| Outcome | *Clinical efficacy*:<br>• The efficacy outcomes were recurrent DVT and PE.<br>• The secondary efficacy outcomes included distal or superficial vein thrombosis and nonfatal or fatal myocardial infarction, stroke, and acute ischemia of the lower limbs. Fatal or nonfatal symptomatic recurrent venous thromboembolism (VTE).<br>*Safety*:<br>• The primary safety outcomes are major or clinically relevant nonmajor bleeding. |

Abbreviations: DOAC, direct oral anticoagulant; PE, pulmonary embolism; VKA, vitamin K antagonist.
Note: PICO stands for P: Population; I: Intervention; C: Comparison or Control; O: Outcome.

**Table 2** Search string on MEDLINE

| Term | Field |
|---|---|
| PE | Title/Abstract |
| DVT | Title/Abstract |
| Venous thromboembolism | Title/Abstract |
| Venous thrombosis | Title/Abstract |
| Embolism | MeSHterms |
| Embolism | Title/Abstract |
| Thrombosis | MeSHterms |
| Embolisms | Title/Abstract |
| Embolus | Title/Abstract |
| Emboli | Title/Abstract |
| Thromboembolism | Title/Abstract |
| Thromboembolisms | Title/Abstract |
| Lung | Title/Abstract |
| Lung | Title/Abstract |
| Lungs | Title/Abstract |
| Pulmonary | Title/Abstract |
| Venous | Title/Abstract |
| Deep vein | Title/Abstract |
| Deepvein | Title/Abstract |
| Deep venous | Title/Abstract |
| Deepvenous | Title/Abstract |
| Anticoagulants | MeSHterms |
| Anticoagulant | Title/Abstract |
| Anticoagulants | Title/Abstract |
| Anticoagulants | Pharmacological action |
| Acenocoumarol | Title/Abstract |

(*Continued*)

**Table 2** (*Continued*)

| Term | Field |
|---|---|
| Apixaban | Title/Abstract |
| Antivitamins K | Title/Abstract |
| Vitamin K | Title/Abstract |
| NOAC | Title/Abstract |
| NOACs | Title/Abstract |
| DOACs | Title/Abstract |
| DOAC | Title/Abstract |
| Warfarin | Title/Abstract |
| Dabigatran | Title/Abstract |
| Edoxaban | Title/Abstract |
| Heparin | Title/Abstract |
| Rivaroxaban | Title/Abstract |
| Aspirin | Title/Abstract |
| Sulodexide | Title/Abstract |
| Acetylsalicylic acid (ASA) | Title/Abstract |
| ASA | Title/Abstract |
| Vessel | Title/Abstract |
| Factor Xa Inhibitors | Pharmacological action |
| Antithrombins | Pharmacological action |
| Heparin | MeSHterms |
| Recurrent | Title/Abstract |
| Recurrence | Title/Abstract |

Abbreviations: DOAC, direct oral anticoagulant; DVT, deep vein thrombosis; MeSH, medical subject heading; NOAC, novel oral anticoagulant; PE, pulmonary embolism.

**Table 3** Allocation of drugs to classes and specific doses modeled as different in the hierarchical Bayesian NMA

| Drug class | Specific drug | Dose |
|---|---|---|
| Placebo | Placebo | N/A |
| Sulodexide | Sulodexide | N/A |
| DOAC | Apixaban | 2.5 mg |
| DOAC | Apixaban | 5 mg |
| DOAC | Dabigatran | N/A |
| DOAC | Edoxaban | N/A |
| DOAC | Rivaroxaban | 10 mg |
| DOAC | Rivaroxaban | 20 mg |
| VKA | Acenocoumarol | N/A |
| VKA | Enoxaparin | N/A |
| VKA | Enoxaparin plus warfarin | N/A |
| VKA | Warfarin | N/A |
| VKA | "VKA regimen" | N/A |
| Aspirin | Aspirin | N/A |

Abbreviations: DOAC, direct oral anticoagulants;NMA, network meta-analysis; VKA, vitamin K antagonist.

**Table 4** Comparison of DICs from fixed effects analysis and consistency models for each outcome, with comment

| Outcome | DIC, analysis model | DIC, consistency model | Comment |
|---|---|---|---|
| DVT | 232.5 | 223.3 | Evidence of inconsistency; direct and indirect evidence appear to disagree |
| PE | 194.8 | 190.9 | Some apparent inconsistency |
| MI | 75.0 | 70.6 | Some apparent inconsistency |
| Stroke | 71.1 | 73.4 | |
| Ischemia | 13.1 | 13.3 | |
| Major bleeding | 168.8 | 171.2 | |
| Other bleeding | 209.2 | 210.7 | |
| Death from VT, MI, stroke | 150.1 | 150.4 | |
| Death from CVD | 68.2 | 67.9 | |
| Death from bleeding | 66.1 | 66.6 | |
| Death from any cause | 175.5 | 176.5 | |

Abbreviations: CVD, cardiovascular disease; DIC, deviance information criterion; DVT, deep venous thrombosis; MI, myocardial infarction; PE, pulmonary embolism; VT, ventricular tachycardia.

**Table 5** Pairwise odds ratios for DVT incidence from consistency and standard fixed-effects model, where the odds ratios estimated from the consistency model were relatively precise

| Comparison | OR, consistency model (95% CI) | OR, standard fixed-effects model (95% CI) | Comment |
|---|---|---|---|
| Sulodexide vs.placebo | 0.45 (0.22–1.00) | 0.50 (0.22–1.00) | Similar |
| DOAC vsplacebo | 0.11 (0.06–0.20) | 0.22 (0.18–0.30) | Direct evidence suggests a stronger effect than indirect evidence |
| VKA vs.placebo | 0.50 (0.37–0.74) | 0.33 (0.25–0.45) | Direct evidence suggests a weaker effect than indirect evidence |
| Aspirin vs.placebo | 0.74 (0.50–1.11) | 0.74 (0.55–1.00) | Similar |
| VKA vs. DOAC | 1.22 (1.00–1.49) | 1.49 (1.11–1.82) | Similar |
| Aspirin vs. DOAC | 3.32 (1.49–7.39) | 3.32 (2.01–4.95) | Similar |

Abbreviations: CI, credible interval; DOAC, direct oral anticoagulants; DVT, deep vein thrombosis; OR, odds ratio; VKA, vitamin K antagonist.

**Table 6** Results of mixed-effects logistic regressions exploring whether age and sex differences were reasonably identifiable with the available data

| Outcome | Factor | Log oddsratio | Std. error | *p*-Value |
|---|---|---|---|---|
| DVT incidence | 1-y change in average age | −0.009 | 0.036 | 0.89 |
| | 10%-point increase in percent male | −0.24 | 0.20 | 0.22 |
| | 1-y increase in follow-up | 0.61 | 0.13 | <0.001 |
| | Receive active treatment (vs. placebo) | −0.82 | 0.096 | <0.001 |
| PE incidence | 1-y change in average age | 0.030 | 0.035 | 0.39 |
| | 10%-point increase in percent male | −0.023 | 0.17 | 0.89 |
| | 1-y increase in follow-up | 0.51 | 0.11 | <0.001 |
| | Receive active treatment (vs. placebo) | −0.68 | 0.13 | <0.001 |
| Major bleeding | 1-y change in average age | 0.030 | 0.049 | 0.54 |
| | 10%-point increase in percent male | −0.18 | 0.25 | 0.48 |
| | 1-y increase in follow-up | 0.23 | 0.18 | 0.22 |
| | Receive active treatment (vs. placebo) | 0.55 | 0.24 | 0.020 |
| Deaths from any cause | 1-y change in average age | 0.015 | 0.041 | 0.71 |
| | 10%-point increase in percent male | −0.31 | 0.21 | 0.14 |
| | 1-y increase in follow-up | 0.57 | 0.15 | <0.001 |
| | Receive active treatment (vs. placebo) | −0.20 | 0.15 | 0.18 |

Abbreviations: DVT, deep vein thrombosis; PE, pulmonary embolism.
Note: Results reported to two significant figures.

# Supplementary Appendix 2 Network diagrams

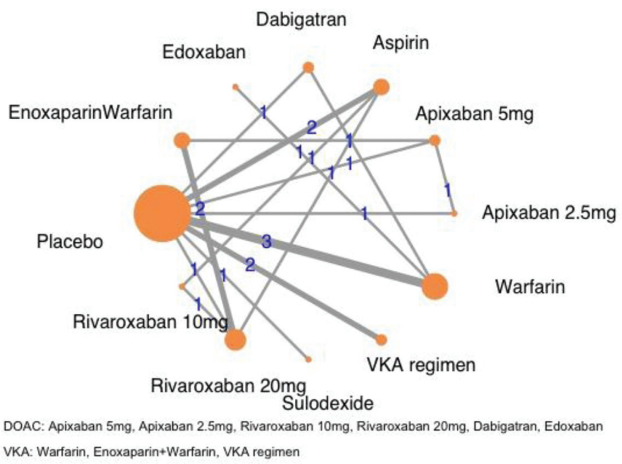


**Fig. 1** Network diagram for evidence on recurrent DVT. DVT, deep vein thrombosis.

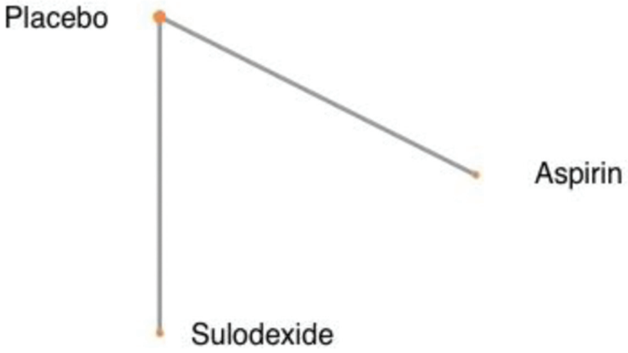


**Fig. 3** Network diagram for evidence on distal thrombosis (calf veins) and superficial VT events. VT, ventral tachycardia.

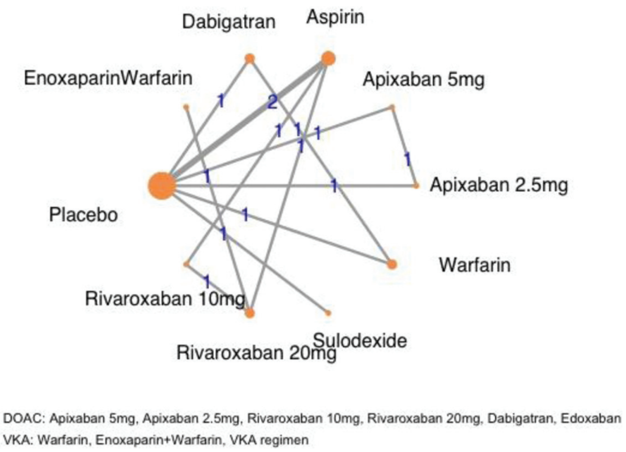


**Fig. 4** Network diagram for evidence on myocardial infarctions.

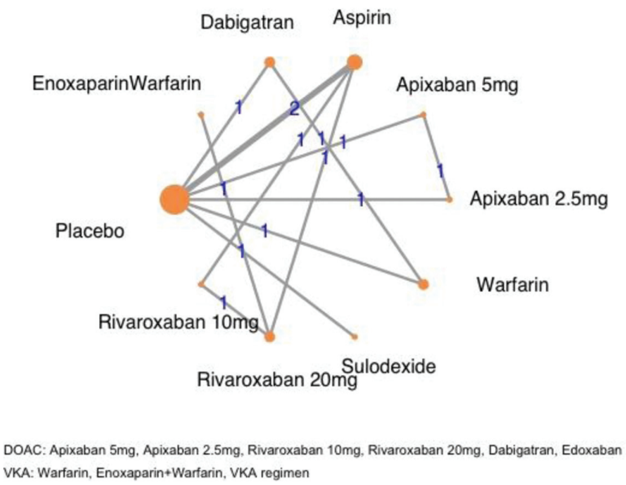


**Fig. 5** Network diagram for evidence on stroke.

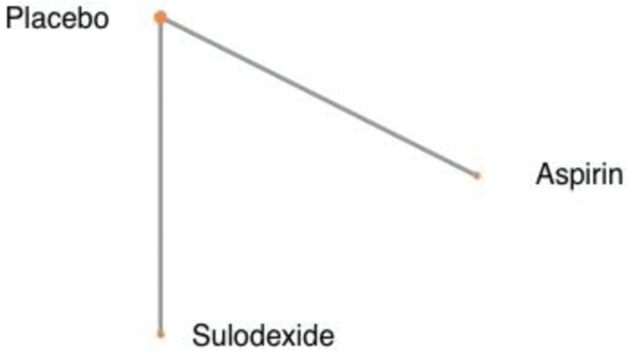


**Fig. 6** Network diagram for evidence on acute ischemia of the lower limbs.

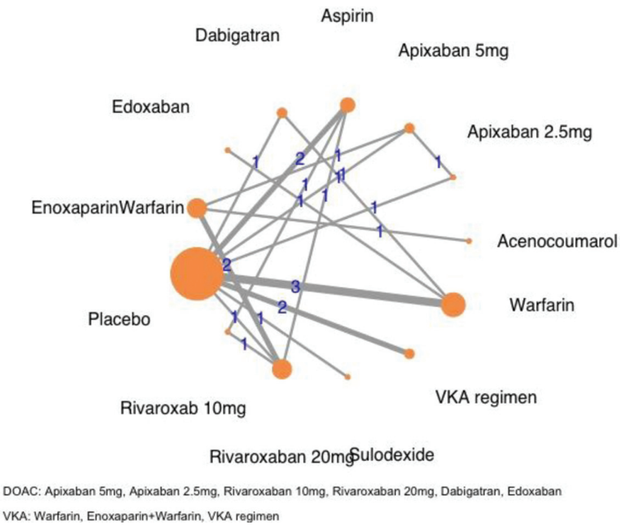


**Fig. 7** Network diagram for evidence on major bleeding.

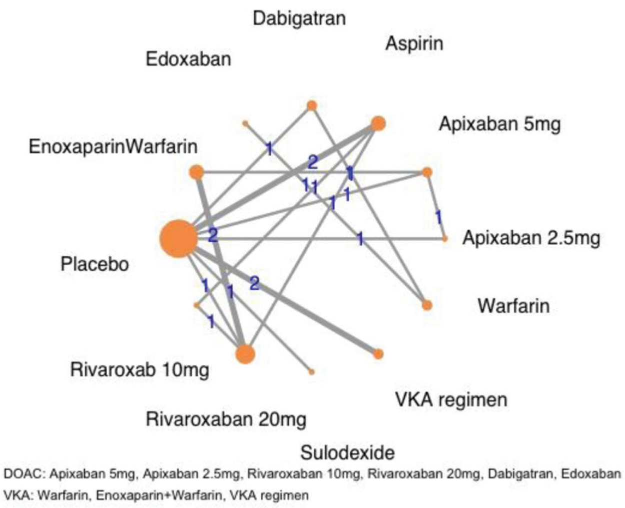


**Fig. 8** Network diagram for evidence on clinically relevant nonmajor bleeding.

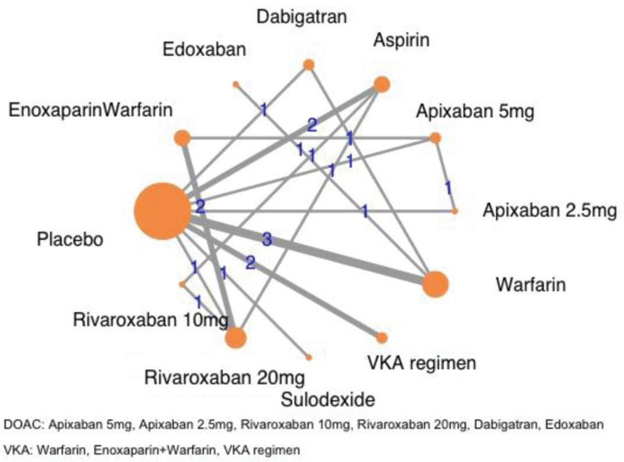


**Fig. 9** Network diagram for evidence on death from VT/PE/MI/stroke. MI, myocardial infarction; PE, pulmonary embolism; VT, ventricular tachycardia.

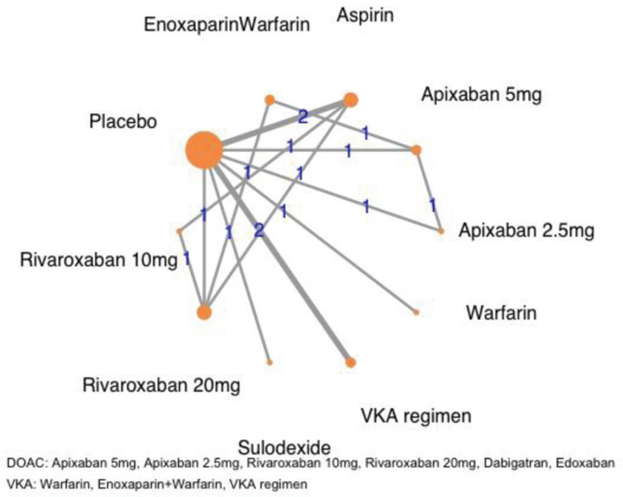


**Fig. 10** Network diagram for evidence on death from CVD. CVD, cardiovascular disease.

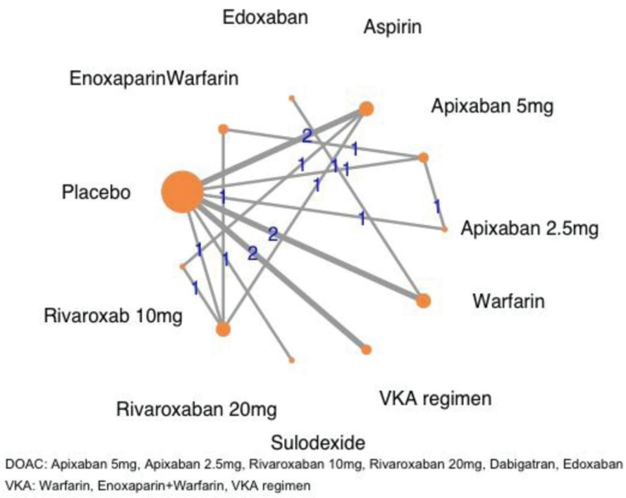


**Fig. 11** Network diagram for evidence on death from bleeding.

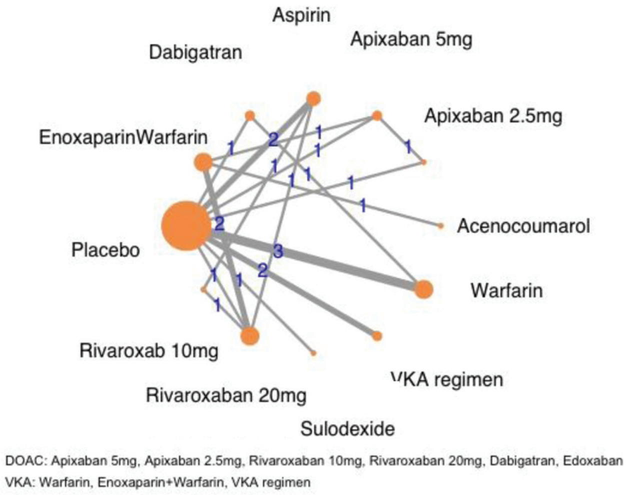


**Fig. 12** Network diagram for evidence on death from any cause (unspecified).

**Supplementary Appendix 3** Baseline characteristics of RCTs and observational studies included in the meta-analysis

| Authors (y) | Treatment arm | Number of patients | Gender (%M) | Age (mean years) | Age (SD years) | Weight (kg) | Weight (SD) | Body mass index (mean) | Body mass index (SD) | Index event is DVT (%) | Index event is PE (%) | Index event is PE with DVT (%) |
|---|---|---|---|---|---|---|---|---|---|---|---|---|
| Raskob et al, 2016 (RCT) | Apixaban | 2,609 | 58.3% | 57.2 | 16.0 | 84.6 | 19.8 | Not provided | Not provided | 65.0% | 25.2% | 9.8% |
| | Enoxaparin plus warfarin | 2,635 | 59.1% | 56.7 | 16.0 | 84.6 | 19.8 | Not provided | Not provided | 65.9% | 25.2% | 8.9% |
| Andreozzi et al, 2015 (RCT) | Sulodexide | 307 | 57.0% | 55.7 | 14.1 | Not provided | Not provided | Not provided | Not provided | 92.5% | 7.5% | 0.0% |
| | Placebo | 308 | 50.3% | 55.9 | 14.4 | Not provided | Not provided | Not provided | Not provided | 92.2% | 7.8% | 0.0% |
| Simes et al, 2014 (RCT) | Aspirin | 411 | 55.0% | 55.0 | 16.0 | Not provided | Not provided | Not provided | Not provided | 57.4% | 27.3% | 15.3% |
| | Placebo | 411 | 53.8% | 54.0 | 15.8 | Not provided | Not provided | Not provided | Not provided | 56.4% | 29.0% | 15.6% |
| Brighton et al, 2012 (RCT) | Aspirin | 411 | 55.0% | 55.0 | 16.0 | Not provided | Not provided | Not provided | Not provided | 57.4% | 27.3% | 15.3% |
| | Placebo | 411 | 53.8% | 54.0 | 15.8 | Not provided | Not provided | Not provided | Not provided | 56.4% | 29.0% | 15.6% |
| Becattini et al, 2012 (RCT) | Aspirin | 205 | 65.8% | 61.9 | 15.3 | Not provided | Not provided | 27.1 | 4.0 | 59.5% | 40.5% | 0.0% |
| | Placebo | 197 | 61.9% | 62.1 | 15.1 | Not provided | Not provided | 27.5 | 3.8 | 65.9% | 34.1% | 0.0% |
| Agnelli et al, 2003 (RCT) | VKA regimen | 165 | 39.4% | 62.9 | 16.3 | Not provided | Not provided | Not provided | Not provided | Not provided | Not provided | Not provided |
| | No treatment | 161 | 41.6% | 61.0 | 15.5 | Not provided | Not provided | Not provided | Not provided | Not provided | Not provided | Not provided |
| Agnelli et al, 2001 (RCT) | VKA regimen | 134 | 54.5% | 66.8 | 6.7 | Not provided | Not provided | Not provided | Not provided | Not provided | Not provided | Not provided |
| | No treatment | 133 | 61.2% | 67.7 | 7.3 | Not provided | Not provided | Not provided | Not provided | Not provided | Not provided | Not provided |
| Kearon et al, 1999 (RCT) | Warfarin | 79 | 68.4% | 59.0 | 16.0 | Not provided | Not provided | Not provided | Not provided | 75.9% | Not provided | Not provided |
| | Placebo | 83 | 53.0% | 58.0 | 16.0 | Not provided | Not provided | Not provided | Not provided | 73.5% | Not provided | Not provided |
| Ridker et al, 2003 (RCT) | Warfarin | 255 | 52.9% | 53.0 | Not provided | Not provided | Not provided | 29.9 | Not provided | Not provided | Not provided | Not provided |
| | Placebo | 253 | 52.6% | 53.0 | Not provided | Not provided | Not provided | 29.9 | Not provided | Not provided | Not provided | Not provided |
| Schulman et al, 2013 (RCT) | Dabigatran | 1,430 | 60.9% | 55.4 | 15.0 | 86.1 | 19.3 | Not provided | Not provided | 65.6% | 22.7% | 11.7% |
| | Warfarin | 1,426 | 61.1% | 53.9 | 15.3 | 86.0 | 18.9 | Not provided | Not provided | 64.7% | 23.5% | 11.8% |
| Schulman et al, 2013 (RCT) | Dabigatran | 681 | 55.0% | 56.1 | 15.5 | 83.7 | 18.0 | Not provided | Not provided | 63.3% | 26.9% | 6.9% |
| | Placebo | 662 | 55.9% | 55.5 | 15.1 | 84.0 | 18.6 | Not provided | Not provided | 66.6% | 26.9% | 5.3% |
| Couturaud et al, 2015 (RCT) | Warfarin | 184 | 42.4% | 58.7 | 17.9 | Not provided | Not provided | 27.8 | 5.9 | Not provided | Not provided | Not provided |
| | Placebo | 187 | 55.1% | 57.3 | 17.4 | Not provided | Not provided | 27.1 | 5.1 | Not provided | Not provided | Not provided |
| Agnelli et al, 2013 (RCT) | Apixaban | 2,609 | 58.3% | 57.2 | 16.0 | 84.6 | 19.8 | Not provided | Not provided | 65.0% | 25.2% | 9.8% |
| | Enoxaparin plus warfarin | 2,635 | 59.1% | 56.7 | 16.0 | 84.6 | 19.8 | Not provided | Not provided | 65.9% | 25.2% | 8.9% |
| Agnelli et al, 2013 (RCT) | Apixaban | 840 | 58.0% | 56.6 | 15.3 | 85.7 | 19.8 | Not provided | Not provided | 64.8% | 35.2% | Not provided |
| | Apixaban | 813 | 57.7% | 56.4 | 15.6 | 85.7 | 19.1 | Not provided | Not provided | 64.8% | 35.2% | Not provided |
| | Placebo | 829 | 56.5% | 57.1 | 15.2 | 84.7 | 18.6 | Not provided | Not provided | 66.5% | 33.5% | Not provided |
| Bauersachs et al, 2010 (RCT) | Rivaroxaban | 1,731 | 57.4% | 55.8 | 16.4 | Not provided | Not provided | Not provided | Not provided | 98.7% | 1.3% | 0.0% |
| | Enoxaparin plus warfarin | 1,718 | 56.3% | 56.4 | 16.3 | Not provided | Not provided | Not provided | Not provided | 98.8% | 1.2% | 0.0% |

(*Continued*)

**Supplementary Appendix 3** (*Continued*)

| Authors (y) | Treatment arm | Number of patients | Gender (%M) | Age (mean years) | Age (SD years) | Weight (kg) | Weight (SD) | Body mass index (mean) | Body mass index (SD) | Index event is DVT (%) | Index event is PE (%) | Index event is PE with DVT (%) |
|---|---|---|---|---|---|---|---|---|---|---|---|---|
| Büller et al, 2012 (RCT) | Rivaroxaban | 2,419 | 54.1% | 57.9 | 7.3 | Not provided | Not provided | Not provided | Not provided | 0.0% | 74.9% | 25.1% |
| | Enoxaparin plus warfarin | 2,413 | 51.7% | 57.5 | 7.2 | Not provided | Not provided | Not provided | Not provided | 0.0% | 75.5% | 24.5% |
| Weitz et al, 2017 (RCT) | Rivaroxaban | 1,107 | 54.4% | 57.9 | 14.7 | Not provided | Not provided | Not provided | Not provided | 51.0% | 34.4% | 14.0% |
| | Rivaroxaban | 1,127 | 55.0% | 58.8 | 14.7 | Not provided | Not provided | Not provided | Not provided | 50.0% | 33.8% | 15.9% |
| | Aspirin | 1,131 | 56.9% | 58.0 | 14.7 | Not provided | Not provided | Not provided | Not provided | 51.0% | 32.4% | 16.0% |
| Romualdi et al 2011 (RCT) | Rivaroxaban | 602 | 58.8% | 58.2 | 15.6 | Not provided | Not provided | Not provided | Not provided | 64.1% | 35.9% | Not provided |
| | Placebo | 594 | 57.1% | 58.4 | 16.0 | Not provided | Not provided | Not provided | Not provided | 59.9% | 40.1% | Not provided |
| Keller et al, 2018 (OBS) | Rivaroxaban | 418 | 48.3% | 60.8 | 17.2 | Not provided | Not provided | Not provided | Not provided | Not provided | Not provided | Not provided |
| Nordstrom et al, 2015 (OBS) | Warfarin | 1,753 | 49.3% | Not provided | Not provided | Not provided | Not provided | Not provided | Not provided | 49.5% | 50.5% | Not provided |
| Douketis et al, 2007 (OBS) | VKA regimen | 2,052 | 47.3% | 62.0 | Not provided | Not provided | Not provided | Not provided | Not provided | 70.7% | 15.1% | 14.2% |
| Castro et al, 2007 (OBS) | Enoxaparin | 181 | 41.4% | 69.0 | 16.0 | 68.0 | 13.0 | Not provided | Not provided | Not provided | Not provided | Not provided |
| | Acenocoumarol | 199 | 49.2% | 67.0 | 16.0 | 70 | 12 | Not provided | Not provided | Not provided | Not provided | Not provided |
| Young et al, 2006 (OBS) | Warfarin | 316 | 44.3% | 55.0 | Not provided | Not provided | Not provided | Not provided | Not provided | Not provided | Not provided | Not provided |
| Schulman et al, 2006 (OBS) | VKA regimen | 897 | Not provided | Not provided | Not provided | Not provided | Not provided | Not provided | Not provided | Not provided | Not provided | Not provided |
| Jiménez et al, 2006 (OBS) | Acenocoumarol | 91 | 53.8% | 69.2 | 14.4 | Not provided | Not provided | 25.6 | 4.4 | 69.2% | 0.0% | 30.8% |

Abbreviations: DVT, deep vein thrombosis; OBS, observational study; PE, pulmonary embolism; RCT, randomized controlled trial; SD, standard deviation; VKA, vitamin K antagonist.

# Supplementary Appendix 4

**Table 1** Drug-specific results for r-DVT from the hierarchical model

| DVT—drug-specific results from hierarchical model—all studies | | | | | | |
|---|---|---|---|---|---|---|
| **Drug** | **Estimate** | **LB** | **UB** | **P(best)** | **SUCRA** | **Average rank** |
| Placebo | 1 | | | 0 | 0.03 | 13.67 |
| Sulodexide | 0.51 | 0.16 | 1.72 | 0.02 | 0.26 | 10.60 |
| DOAC—Apixaban—2.5mg | 0.18 | 0.08 | 0.32 | 0.22 | 0.83 | 3.22 |
| DOAC—Apixaban—5mg | 0.19 | 0.09 | 0.38 | 0.18 | 0.78 | 3.80 |
| DOAC—Dabigatran | 0.2 | 0.1 | 0.39 | 0.11 | 0.73 | 4.49 |
| DOAC—Edoxaban | 0.21 | 0.1 | 0.4 | 0.09 | 0.71 | 4.73 |
| DOAC—Rivaroxaban—10mg | 0.2 | 0.1 | 0.35 | 0.1 | 0.75 | 4.23 |
| DOAC—Rivaroxaban—20mg | 0.19 | 0.08 | 0.37 | 0.23 | 0.79 | 3.67 |
| VKA—Acenocoumarol | 0.35 | 0.14 | 0.92 | 0.02 | 0.4 | 8.81 |
| VKA—Enoxaparin | 0.35 | 0.14 | 0.93 | 0.02 | 0.4 | 8.84 |
| VKA—Enoxaparin plus warfarin | 0.31 | 0.16 | 0.61 | 0 | 0.44 | 8.24 |
| VKA—Warfarin | 0.29 | 0.16 | 0.5 | 0 | 0.5 | 7.53 |
| VKA—“VKA regimen” | 0.48 | 0.25 | 1.01 | 0 | 0.24 | 10.89 |
| Aspirin | 0.71 | 0.35 | 1.27 | 0 | 0.13 | 12.26 |

Abbreviations: DOAC, direct-acting oral anticoagulant; LB, lower bound; r-DVT, recurrent deep venous thrombosis; P, probability;SUCRA, surface under the cumulative ranking curve; UB, upper bound; VKA, vitamin K antagonist.

**Table 2** Drug-specific results for PE from the hierarchical model

| PE—drug-specific results from hierarchical model—all studies | | | | | | |
|---|---|---|---|---|---|---|
| **Drug** | **Estimate** | **LB** | **UB** | **P(best)** | **SUCRA** | **Average rank** |
| Placebo | 1 | | | 0 | 0.04 | 13.52 |
| Sulodexide | 0.43 | 0.11 | 2.21 | 0.2 | 0.47 | 7.90 |
| DOAC—Apixaban—2.5mg | 0.36 | 0.19 | 0.66 | 0.03 | 0.58 | 6.46 |
| DOAC—Apixaban—5mg | 0.31 | 0.14 | 0.61 | 0.16 | 0.71 | 4.78 |
| DOAC—Dabigatran | 0.32 | 0.17 | 0.64 | 0.1 | 0.67 | 5.35 |
| DOAC—Edoxaban | 0.33 | 0.15 | 0.66 | 0.1 | 0.67 | 5.34 |
| DOAC—Rivaroxaban—10mg | 0.33 | 0.18 | 0.58 | 0.05 | 0.66 | 5.45 |
| DOAC—Rivaroxaban—20mg | 0.31 | 0.14 | 0.63 | 0.15 | 0.71 | 4.74 |
| VKA—Acenocoumarol | 0.41 | 0.17 | 0.98 | 0.06 | 0.48 | 7.80 |
| VKA—Enoxaparin | 0.42 | 0.17 | 0.96 | 0.06 | 0.48 | 7.78 |
| VKA—Enoxaparin plus warfarin | 0.35 | 0.18 | 0.65 | 0.05 | 0.6 | 6.23 |
| VKA—Warfarin | 0.38 | 0.2 | 0.69 | 0.02 | 0.51 | 7.34 |
| VKA—“VKA regimen” | 0.5 | 0.24 | 1.07 | 0.01 | 0.33 | 9.74 |
| Aspirin | 0.8 | 0.45 | 1.67 | 0 | 0.11 | 12.58 |

Abbreviations: DOAC, direct-acting oral anticoagulant; LB, lower bound; P, probability;PE, pulmonary embolism; SUCRA, surface under the cumulative ranking curve; UB, upper bound; VKA, vitamin K antagonist.

**Table 3** Main results for MI, all studies

| MI—main results—all studies | | | | | | | | | |
|---|---|---|---|---|---|---|---|---|---|
| Statistic | Fixed model estimate | LB | UB | Random model estimate | LB | UB | Hierarchical estimate | LB | UB |
| DIC | 75 | | | 74 | | | 79 | | |
| OR sulodexide vs. placebo | 0.49 | 0.03 | 4.86 | 0.5 | 0.02 | 11.27 | 0.74 | 0.11 | 4.72 |
| OR DOAC vs. placebo | 0.75 | 0.25 | 2.16 | 0.78 | 0.15 | 3.78 | 0.64 | 0.13 | 2.6 |
| OR VKA vs. placebo | 0.27 | 0.07 | 0.96 | 0.31 | 0.04 | 2.81 | 0.33 | 0.04 | 2.88 |
| OR aspirin vs. placebo | 0.78 | 0.29 | 2.27 | 0.87 | 0.17 | 4.56 | 0.93 | 0.21 | 5.39 |
| OR DOACvs.sulodexide | 1.54 | 0.11 | 34.82 | 1.45 | 0.05 | 51.77 | 0.85 | 0.09 | 8.35 |
| OR VKA vs.sulodexide | 0.53 | 0.04 | 12.76 | 0.61 | 0.01 | 28.27 | 0.46 | 0.03 | 8.84 |
| OR aspirin vs.sulodexide | 1.61 | 0.14 | 34.12 | 1.75 | 0.05 | 51.56 | 1.39 | 0.18 | 9.12 |
| OR VKA vs. DOAC | 0.36 | 0.09 | 1.06 | 0.4 | 0.05 | 3.38 | 0.53 | 0.07 | 4.79 |
| OR aspirin vs. DOAC | 1.04 | 0.31 | 3.54 | 1.07 | 0.17 | 8.81 | 1.55 | 0.25 | 10.88 |
| OR aspirin vs. VKA | 2.87 | 0.66 | 15.18 | 2.82 | 0.17 | 29.52 | 3.02 | 0.21 | 36.33 |
| P(placebo best) | 0 | | | 0.01 | | | 0.02 | | |
| P(sulodexide best) | 0.34 | | | 0.34 | | | 0.21 | | |
| P(DOAC best) | 0.01 | | | 0.06 | | | 0.14 | | |
| P(VKA best) | 0.62 | | | 0.52 | | | 0.56 | | |
| P(aspirin best) | 0.03 | | | 0.07 | | | 0.07 | | |
| P(placebo worst) | 0.37 | | | 0.28 | | | 0.29 | | |
| P(sulodexide worst) | 0.22 | | | 0.26 | | | 0.23 | | |
| P(DOAC worst) | 0.2 | | | 0.18 | | | 0.09 | | |
| P(VKA worst) | 0 | | | 0.04 | | | 0.06 | | |
| P(aspirin worst) | 0.21 | | | 0.24 | | | 0.32 | | |
| SUCRA(placebo) | 0.23 | | | 0.3 | | | 0.07 | | |
| SUCRA(sulodexide) | 0.59 | | | 0.57 | | | 0.31 | | |
| SUCRA(DOAC) | 0.41 | | | 0.44 | | | 0.3 | | |
| SUCRA(VKA) | 0.88 | | | 0.8 | | | 0.67 | | |
| SUCRA(aspirin) | 0.4 | | | 0.4 | | | 0.15 | | |
| Average rank (placebo) | 4.09 | | | 3.81 | | | 3.76 | | |
| Average rank (sulodexide) | 2.64 | | | 2.73 | | | 3.03 | | |
| Average rank (DOAC) | 3.38 | | | 3.26 | | | 2.77 | | |
| Average rank (VKA) | 1.48 | | | 1.8 | | | 1.86 | | |
| Average rank (aspirin) | 3.4 | | | 3.41 | | | 3.59 | | |

Abbreviations: DIC, deviance information criterion; DOAC, direct-acting oral anticoagulant; LB, lower bound; MI, myocardial infarction; OR, odds ratio; P, probability;SUCRA, surface under the cumulative ranking curve; UB, upper bound; VKA, vitamin K antagonist.

**Table 4** Drug-specific results for MI from the hierarchical model

| MI—drug-specific results from hierarchical model—all studies | | | | | | |
|---|---|---|---|---|---|---|
| Drug | Estimate | LB | UB | P(best) | SUCRA | Average rank |
| Placebo | 1 | | | 0.01 | 0.25 | 10.74 |
| Sulodexide | 0.76 | 0.11 | 4.72 | 0.12 | 0.42 | 8.55 |
| DOAC—Apixaban—2.5mg | 0.59 | 0.12 | 2.73 | 0.03 | 0.48 | 7.82 |
| DOAC—Apixaban—5mg | 0.68 | 0.13 | 2.85 | 0.01 | 0.41 | 8.63 |
| DOAC—Dabigatran | 0.86 | 0.16 | 4.83 | 0 | 0.31 | 9.99 |
| DOAC—Edoxaban | 0.64 | 0.09 | 3.73 | 0.04 | 0.44 | 8.29 |
| DOAC—Rivaroxaban—10mg | 0.59 | 0.09 | 2.65 | 0.05 | 0.49 | 7.66 |
| DOAC—Rivaroxaban—20mg | 0.52 | 0.06 | 2.44 | 0.08 | 0.56 | 6.74 |
| VKA—Acenocoumarol | 0.34 | 0.04 | 4.41 | 0.13 | 0.66 | 5.38 |
| VKA—Enoxaparin | 0.34 | 0.04 | 4.87 | 0.14 | 0.67 | 5.33 |
| VKA—Enoxaparin plus warfarin | 0.32 | 0.04 | 3.85 | 0.12 | 0.66 | 5.42 |
| VKA—Warfarin | 0.32 | 0.06 | 2.11 | 0.09 | 0.69 | 5.09 |
| VKA—“VKA regimen” | 0.32 | 0.04 | 3.56 | 0.15 | 0.68 | 5.19 |
| Aspirin | 0.93 | 0.21 | 5.3 | 0.02 | 0.29 | 10.17 |

Abbreviations: DOAC, direct-acting oral anticoagulant; LB, lower bound; MI, myocardial infarction; P, probability;SUCRA, surface under the cumulative ranking curve; UB, upper bound; VKA, vitamin K antagonist.

**Table 5** Main results for stroke, all studies

| Stroke—main results—all studies | | | | | | | | | |
|---|---|---|---|---|---|---|---|---|---|
| Statistic | Fixed model estimate | LB | UB | Random model estimate | LB | UB | Hierarchical estimate | LB | UB |
| DIC | 71 | | | 72 | | | 79 | | |
| OR sulodexide vs. placebo | 6.75 | 0.18 | 1370.08 | 5.99 | 0.23 | 1363.11 | 8.97 | 0.2 | 1060.88 |
| OR DOAC vs. placebo | 0.43 | 0.14 | 1.2 | 0.44 | 0.13 | 1.39 | 0.49 | 0.15 | 1.5 |
| OR VKA vs. placebo | 0.91 | 0.2 | 3.61 | 0.85 | 0.13 | 4.23 | 0.91 | 0.16 | 5.37 |
| OR aspirin vs. placebo | 0.82 | 0.28 | 2.32 | 0.81 | 0.23 | 3.07 | 0.61 | 0.17 | 2.15 |
| OR DOAC vs.sulodexide | 0.06 | 0 | 2.87 | 0.08 | 0 | 2.44 | 0.05 | 0 | 2.49 |
| OR VKA vs.sulodexide | 0.13 | 0 | 8.54 | 0.13 | 0 | 5.19 | 0.07 | 0 | 6.5 |
| OR aspirin vs.sulodexide | 0.11 | 0 | 5.9 | 0.13 | 0 | 4.22 | 0.06 | 0 | 3.34 |
| OR VKA vs. DOAC | 2.14 | 0.69 | 7.47 | 1.96 | 0.41 | 8.48 | 1.86 | 0.34 | 11.81 |
| OR aspirin vs. DOAC | 1.86 | 0.53 | 7.99 | 1.83 | 0.46 | 8.62 | 1.28 | 0.32 | 5.19 |
| OR aspirin vs. VKA | 0.92 | 0.17 | 5.66 | 0.97 | 0.15 | 7.15 | 0.67 | 0.12 | 4.8 |
| P(placebo best) | 0.03 | | | 0.03 | | | 0.02 | | |
| P(sulodexide best) | 0.08 | | | 0.06 | | | 0.07 | | |
| P(DOAC best) | 0.67 | | | 0.6 | | | 0.47 | | |
| P(VKA best) | 0.09 | | | 0.15 | | | 0.16 | | |
| P(aspirin best) | 0.13 | | | 0.16 | | | 0.27 | | |
| P(placebo worst) | 0.06 | | | 0.06 | | | 0.05 | | |
| P(sulodexide worst) | 0.78 | | | 0.77 | | | 0.84 | | |
| P(DOAC worst) | 0 | | | 0.01 | | | 0 | | |
| P(VKA worst) | 0.09 | | | 0.1 | | | 0.08 | | |
| P(aspirin worst) | 0.06 | | | 0.07 | | | 0.02 | | |
| SUCRA(placebo) | 0.43 | | | 0.43 | | | 0.08 | | |
| SUCRA(sulodexide) | 0.14 | | | 0.14 | | | 0.08 | | |

(*Continued*)

**Table 5** (*Continued*)

| Stroke—main results—all studies | | | | | | | | | |
|---|---|---|---|---|---|---|---|---|---|
| Statistic | Fixed model estimate | LB | UB | Random model estimate | LB | UB | Hierarchical estimate | LB | UB |
| SUCRA(DOAC) | 0.9 | | | 0.86 | | | 0.64 | | |
| SUCRA(VKA) | 0.49 | | | 0.52 | | | 0.25 | | |
| SUCRA(aspirin) | 0.54 | | | 0.55 | | | 0.44 | | |
| Average rank (placebo) | 3.29 | | | 3.29 | | | 3.38 | | |
| Average rank (sulodexide) | 4.42 | | | 4.45 | | | 4.55 | | |
| Average rank (DOAC) | 1.42 | | | 1.55 | | | 1.77 | | |
| Average rank (VKA) | 3.04 | | | 2.92 | | | 3.01 | | |
| Average rank (aspirin) | 2.83 | | | 2.8 | | | 2.3 | | |

Abbreviations: DIC, deviance information criterion;DOAC, direct-acting oral anticoagulant; LB, lower bound; OR, odds ratio; P, probability;SUCRA, surface under the cumulative ranking curve; UB, upper bound; VKA, vitamin K antagonist.

**Table 6** Drug-specific results for stroke from the hierarchical model

| Stroke—drug-specific results from hierarchical model—all studies | | | | | | |
|---|---|---|---|---|---|---|
| Drug | Estimate | LB | UB | P(best) | SUCRA | Average rank |
| Placebo | 1 | | | 0 | 0.32 | 9.87 |
| Sulodexide | 8.91 | 0.2 | 1097.46 | 0.04 | 0.11 | 12.59 |
| DOAC—Apixaban—2.5mg | 0.44 | 0.11 | 1.48 | 0.13 | 0.72 | 4.68 |
| DOAC—Apixaban—5mg | 0.44 | 0.11 | 1.53 | 0.15 | 0.72 | 4.59 |
| DOAC—Dabigatran | 0.57 | 0.15 | 2.28 | 0.07 | 0.59 | 6.37 |
| DOAC—Edoxaban | 0.49 | 0.11 | 2.17 | 0.12 | 0.66 | 5.41 |
| DOAC—Rivaroxaban—10mg | 0.44 | 0.12 | 1.6 | 0.1 | 0.72 | 4.64 |
| DOAC—Rivaroxaban—20mg | 0.57 | 0.15 | 2.25 | 0.05 | 0.59 | 6.32 |
| VKA—Acenocoumarol | 0.91 | 0.1 | 7.13 | 0.05 | 0.41 | 8.70 |
| VKA—Enoxaparin | 0.9 | 0.14 | 7.15 | 0.04 | 0.41 | 8.73 |
| VKA—Enoxaparin plus warfarin | 0.95 | 0.17 | 5.04 | 0.01 | 0.37 | 9.21 |
| VKA—Warfarin | 0.86 | 0.15 | 4.73 | 0.04 | 0.43 | 8.40 |
| VKA—“VKA regimen” | 0.9 | 0.12 | 6.69 | 0.05 | 0.41 | 8.65 |
| Aspirin | 0.6 | 0.17 | 2.19 | 0.13 | 0.55 | 6.84 |

Abbreviations: DOAC, direct-acting oral anticoagulant; LB, lower bound; P, probability; SUCRA, surface under the cumulative ranking curve; UB, upper bound; VKA, vitamin K antagonist.

**Table 7** Drug-specific results for major bleeding from the hierarchical model

| Major bleed—drug-specific results from hierarchical model—all studies | | | | | | |
|---|---|---|---|---|---|---|
| Drug | Estimate | LB | UB | P(best) | SUCRA | Average rank |
| Placebo | 1 | | | 0.1 | 0.82 | 3.30 |
| Sulodexide | 0.19 | 0 | 10.62 | 0.71 | 0.8 | 3.59 |
| DOAC—Apixaban—2.5mg | 1.32 | 0.53 | 3.21 | 0.03 | 0.71 | 4.83 |
| DOAC—Apixaban—5mg | 1.34 | 0.51 | 3.49 | 0.03 | 0.67 | 5.23 |
| DOAC—Dabigatran | 1.49 | 0.67 | 3.86 | 0.01 | 0.6 | 6.17 |
| DOAC—Edoxaban | 1.75 | 0.74 | 4.27 | 0.01 | 0.51 | 7.32 |
| DOAC—Rivaroxaban—10mg | 1.79 | 0.82 | 4.01 | 0 | 0.49 | 7.63 |
| DOAC—Rivaroxaban—20mg | 1.53 | 0.65 | 3.8 | 0.01 | 0.59 | 6.38 |
| VKA—Acenocoumarol | 2.83 | 1.07 | 7.73 | 0 | 0.21 | 11.29 |
| VKA—Enoxaparin | 2.75 | 1.03 | 7.08 | 0 | 0.23 | 11.06 |
| VKA—Enoxaparin plus warfarin | 3.17 | 1.39 | 7.36 | 0 | 0.13 | 12.32 |
| VKA—Warfarin | 2.51 | 1.33 | 5.75 | 0 | 0.25 | 10.78 |
| VKA—“VKA regimen” | 2.73 | 1.17 | 6.55 | 0 | 0.22 | 11.10 |
| Aspirin | 1.02 | 0.44 | 2.56 | 0.1 | 0.77 | 4.00 |

Abbreviations: DOAC, direct-acting oral anticoagulant; LB, lower bound; P, probability;SUCRA, surface under the cumulative ranking curve; UB, upper bound; VKA, vitamin K antagonist.

**Table 8** Drug-specific results for clinically relevant nonmajor bleeding from the hierarchical model

| Other bleeding—drug-specific results from hierarchical model—all studies | | | | | | |
|---|---|---|---|---|---|---|
| Drug | Estimate | LB | UB | P(best) | SUCRA | Average rank |
| Placebo | 1 | | | 0.3 | 0.94 | 1.82 |
| Sulodexide | 0.59 | 0.02 | 3.67 | 0.67 | 0.88 | 2.55 |
| DOAC—Apixaban—2.5mg | 1.82 | 0.99 | 3.41 | 0.01 | 0.72 | 4.58 |
| DOAC—Apixaban—5mg | 2.28 | 1.23 | 4.15 | 0 | 0.53 | 7.05 |
| DOAC—Dabigatran | 2.21 | 1.26 | 4 | 0 | 0.57 | 6.59 |
| DOAC—Edoxaban | 2.51 | 1.23 | 5.04 | 0 | 0.48 | 7.78 |
| DOAC—Rivaroxaban—10mg | 2.99 | 1.59 | 5.14 | 0 | 0.3 | 10.16 |
| DOAC—Rivaroxaban—20mg | 2.23 | 1.16 | 4.51 | 0 | 0.55 | 6.89 |
| VKA—Acenocoumarol | 3.08 | 1.14 | 7.58 | 0 | 0.3 | 10.08 |
| VKA—Enoxaparin | 3.04 | 1.24 | 7.86 | 0 | 0.29 | 10.25 |
| VKA—Enoxaparin plus warfarin | 3.2 | 1.74 | 5.83 | 0 | 0.23 | 11.01 |
| VKA—Warfarin | 3.37 | 1.68 | 6.52 | 0 | 0.2 | 11.36 |
| VKA—“VKA regimen” | 2.85 | 1.2 | 7.01 | 0 | 0.35 | 9.50 |
| Aspirin | 1.88 | 0.89 | 4.19 | 0.01 | 0.66 | 5.38 |

Abbreviations: DOAC, direct-acting oral anticoagulant; LB, lower bound; P, probability;SUCRA, surface under the cumulative ranking curve; UB, upper bound; VKA, vitamin K antagonist.

**Table 9** Drug-specific results for death from VTE/PE/MI/stroke from the hierarchical model

| Death from VTE, MI, stroke—drug-specific results from hierarchical model—all studies | | | | | | |
|---|---|---|---|---|---|---|
| **Drug** | **Estimate** | **LB** | **UB** | **P(best)** | **SUCRA** | **Average rank** |
| Placebo | 1 | | | 0 | 0.23 | 11.00 |
| Sulodexide | 0.28 | 0.02 | 2.15 | 0.57 | 0.77 | 3.97 |
| DOAC—Apixaban—2.5mg | 0.5 | 0.15 | 1.29 | 0.08 | 0.7 | 4.90 |
| DOAC—Apixaban—5mg | 0.56 | 0.19 | 1.61 | 0.04 | 0.62 | 5.92 |
| DOAC—Dabigatran | 0.53 | 0.15 | 1.5 | 0.06 | 0.66 | 5.38 |
| DOAC—Edoxaban | 0.6 | 0.19 | 1.77 | 0.03 | 0.56 | 6.77 |
| DOAC—Rivaroxaban—10mg | 0.66 | 0.23 | 1.61 | 0.02 | 0.49 | 7.57 |
| DOAC—Rivaroxaban—20mg | 0.5 | 0.13 | 1.5 | 0.1 | 0.68 | 5.13 |
| VKA—Acenocoumarol | 0.74 | 0.22 | 2.39 | 0.03 | 0.42 | 8.52 |
| VKA—Enoxaparin | 0.72 | 0.22 | 2.49 | 0.02 | 0.43 | 8.37 |
| VKA—Enoxaparin plus warfarin | 0.67 | 0.26 | 1.7 | 0.01 | 0.48 | 7.78 |
| VKA—Warfarin | 0.71 | 0.27 | 1.86 | 0.02 | 0.45 | 8.17 |
| VKA—“VKA regimen” | 0.83 | 0.33 | 2.26 | 0.01 | 0.34 | 9.56 |
| Aspirin | 1.49 | 0.37 | 5.13 | 0 | 0.16 | 11.97 |

Abbreviations: DOAC, direct-acting oral anticoagulant; LB, lower bound; MI, myocardial infarction; P, probability;PE, pulmonary embolism; SUCRA, surface under the cumulative ranking curve; UB, upper bound; VKA, vitamin K antagonist; VTE, venous thromboembolism.

**Table 10** Main results for death from CVD, all studies

| Death from CVD—main results—all studies | | | | | | | | | |
|---|---|---|---|---|---|---|---|---|---|
| **Statistic** | **Fixed model estimate** | **LB** | **UB** | **Random model estimate** | **LB** | **UB** | **Hierarchical estimate** | **LB** | **UB** |
| DIC | 68 | | | 68 | | | 68 | | |
| OR sulodexide vs. placebo | 0.81 | 0 | 434.81 | 0.68 | 0 | 172.35 | 6.01 | 0.07 | 145.46 |
| OR DOAC vs. placebo | 0.31 | 0.07 | 1.36 | 0.27 | 0.02 | 2.28 | 0.19 | 0.01 | 1.39 |
| OR VKA vs. placebo | 0.61 | 0.14 | 2.57 | 0.54 | 0.05 | 3.68 | 0.49 | 0.06 | 2.68 |
| OR aspirin vs. placebo | 0.2 | 0.02 | 1.1 | 0.2 | 0.01 | 5.99 | 0.12 | 0.02 | 0.66 |
| OR DOAC vs.sulodexide | 0.32 | 0 | 209.13 | 0.41 | 0 | 93.49 | 0.03 | 0 | 3.05 |
| OR VKA vs.sulodexide | 0.71 | 0 | 397.04 | 0.72 | 0 | 228.47 | 0.09 | 0 | 8.01 |
| OR aspirin vs.sulodexide | 0.2 | 0 | 91.81 | 0.3 | 0 | 211.84 | 0.03 | 0 | 1.18 |
| OR VKA vs. DOAC | 1.92 | 0.87 | 4.69 | 1.98 | 0.33 | 20.44 | 2.48 | 0.5 | 15.66 |
| OR aspirin vs. DOAC | 0.6 | 0.04 | 6.07 | 0.61 | 0.02 | 75.13 | 0.56 | 0.04 | 13.65 |
| OR aspirin vs. VKA | 0.31 | 0.02 | 2.84 | 0.31 | 0.01 | 24.9 | 0.23 | 0.02 | 3.25 |
| P(placebo best) | 0 | | | 0 | | | 0 | | |
| P(sulodexide best) | 0.28 | | | 0.28 | | | 0.03 | | |
| P(DOAC best) | 0.23 | | | 0.23 | | | 0.32 | | |
| P(VKA best) | 0 | | | 0.03 | | | 0.04 | | |
| P(aspirin best) | 0.48 | | | 0.45 | | | 0.62 | | |
| P(placebo worst) | 0.39 | | | 0.35 | | | 0.16 | | |
| P(sulodexide worst) | 0.47 | | | 0.42 | | | 0.78 | | |
| P(DOAC worst) | 0.01 | | | 0.02 | | | 0.01 | | |
| P(VKA worst) | 0.12 | | | 0.15 | | | 0.05 | | |
| P(aspirin worst) | 0.01 | | | 0.07 | | | 0 | | |

**Table 10** (*Continued*)

| Death from CVD—main results—all studies | | | | | | | | | |
|---|---|---|---|---|---|---|---|---|---|
| Statistic | Fixed model estimate | LB | UB | Random model estimate | LB | UB | Hierarchical estimate | LB | UB |
| SUCRA(placebo) | 0.2 | | | 0.23 | | | 0.01 | | |
| SUCRA(sulodexide) | 0.41 | | | 0.44 | | | 0.06 | | |
| SUCRA(DOAC) | 0.72 | | | 0.69 | | | 0.57 | | |
| SUCRA(VKA) | 0.38 | | | 0.41 | | | 0.12 | | |
| SUCRA(aspirin) | 0.79 | | | 0.73 | | | 0.74 | | |
| Average rank (placebo) | 4.19 | | | 4.08 | | | 3.97 | | |
| Average rank (sulodexide) | 3.35 | | | 3.23 | | | 4.53 | | |
| Average rank (DOAC) | 2.14 | | | 2.23 | | | 1.9 | | |
| Average rank (VKA) | 3.47 | | | 3.36 | | | 3.06 | | |
| Average rank (aspirin) | 1.85 | | | 2.1 | | | 1.53 | | |

Abbreviations: CVD, cardiovascular disease; DIC, deviance information criterion; DOAC, direct-acting oral anticoagulant; LB, lower bound; OR, odds ratio; P, probability;SUCRA, surface under the cumulative ranking curve; UB, upper bound; VKA, vitamin K antagonist.

**Table 11** Drug-specific results for death from CVD from the hierarchical model

| Death from CVD—drug-specific results from hierarchical model—all studies | | | | | | |
|---|---|---|---|---|---|---|
| Drug | Estimate | LB | UB | P(best) | SUCRA | Average rank |
| Placebo | 1 | | | 0 | 0.17 | 11.83 |
| Sulodexide | 6.06 | 0.06 | 145.56 | 0.02 | 0.11 | 12.55 |
| DOAC—Apixaban—2.5mg | 0.18 | 0.02 | 1.35 | 0.08 | 0.72 | 4.62 |
| DOAC—Apixaban—5mg | 0.19 | 0.01 | 1.34 | 0.08 | 0.72 | 4.70 |
| DOAC—Dabigatran | 0.2 | 0.01 | 1.68 | 0.09 | 0.69 | 5.09 |
| DOAC—Edoxaban | 0.2 | 0.01 | 1.62 | 0.07 | 0.68 | 5.11 |
| DOAC—Rivaroxaban—10mg | 0.21 | 0.02 | 1.65 | 0.03 | 0.64 | 5.62 |
| DOAC—Rivaroxaban—20mg | 0.19 | 0.01 | 1.76 | 0.09 | 0.69 | 4.98 |
| VKA—Acenocoumarol | 0.49 | 0.05 | 3.55 | 0.01 | 0.37 | 9.19 |
| VKA—Enoxaparin | 0.47 | 0.06 | 3.12 | 0.01 | 0.37 | 9.24 |
| VKA—Enoxaparin plus warfarin | 0.43 | 0.06 | 2.77 | 0 | 0.39 | 8.97 |
| VKA—Warfarin | 0.49 | 0.05 | 2.79 | 0.02 | 0.37 | 9.19 |
| VKA—“VKA regimen” | 0.57 | 0.08 | 3.17 | 0 | 0.31 | 9.99 |
| Aspirin | 0.11 | 0.02 | 0.66 | 0.49 | 0.77 | 3.93 |

Abbreviations: CVD, cardiovascular disease;DOAC, direct-acting oral anticoagulant; LB, lower bound; P, probability;SUCRA, surface under the cumulative ranking curve; UB, upper bound; VKA, vitamin K antagonist.

**Table 12** Main results for death from bleeding, all studies

| Death from bleeding—main results—all studies | | | | | | | | | |
|---|---|---|---|---|---|---|---|---|---|
| **Statistic** | **Fixed model estimate** | **LB** | **UB** | **Random model estimate** | **LB** | **UB** | **Hierarchical estimate** | **LB** | **UB** |
| DIC | 66 | | | 66 | | | 66 | | |
| OR sulodexide vs. placebo | 0.86 | 0 | 685.67 | 0.85 | 0 | 246.49 | 0.1 | 0 | 7.72 |
| OR DOAC vs. placebo | 0.21 | 0.02 | 1.57 | 0.23 | 0.02 | 2.46 | 0.13 | 0.01 | 1.34 |
| OR VKA vs. placebo | 0.49 | 0.04 | 3.22 | 0.56 | 0.04 | 5.58 | 0.35 | 0.03 | 2.88 |
| OR aspirin vs. placebo | 0.13 | 0.01 | 1.71 | 0.15 | 0 | 2.05 | 0.1 | 0 | 1.07 |
| OR DOAC vs.sulodexide | 0.23 | 0 | 114.15 | 0.3 | 0 | 542.25 | 1 | 0.01 | 184.43 |
| OR VKA vs.sulodexide | 0.49 | 0 | 285.9 | 0.69 | 0 | 1196.34 | 2.76 | 0.03 | 474.67 |
| OR aspirin vs.sulodexide | 0.15 | 0 | 97.8 | 0.2 | 0 | 252.07 | 0.57 | 0 | 169.95 |
| OR VKA vs. DOAC | 2.28 | 1.07 | 5.03 | 2.28 | 0.7 | 8.18 | 2.61 | 0.74 | 9.83 |
| OR aspirin vs. DOAC | 0.65 | 0.04 | 8.96 | 0.66 | 0.02 | 12 | 0.76 | 0.02 | 14.39 |
| OR aspirin vs. VKA | 0.28 | 0.02 | 3.97 | 0.28 | 0.01 | 5.83 | 0.3 | 0.01 | 5.83 |
| P(placebo best) | 0.01 | | | 0.01 | | | 0 | | |
| P(sulodexide best) | 0.24 | | | 0.26 | | | 0.33 | | |
| P(DOAC best) | 0.25 | | | 0.25 | | | 0.25 | | |
| P(VKA best) | 0 | | | 0.02 | | | 0.02 | | |
| P(aspirin best) | 0.49 | | | 0.46 | | | 0.39 | | |
| P(placebo worst) | 0.37 | | | 0.35 | | | 0.61 | | |
| P(sulodexide worst) | 0.46 | | | 0.44 | | | 0.24 | | |
| P(DOAC worst) | 0 | | | 0.01 | | | 0.01 | | |
| P(VKA worst) | 0.14 | | | 0.18 | | | 0.13 | | |
| P(aspirin worst) | 0.03 | | | 0.03 | | | 0.01 | | |
| SUCRA(placebo) | 0.22 | | | 0.24 | | | 0.01 | | |
| SUCRA(sulodexide) | 0.39 | | | 0.41 | | | 0.44 | | |
| SUCRA(DOAC) | 0.74 | | | 0.72 | | | 0.44 | | |
| SUCRA(VKA) | 0.38 | | | 0.37 | | | 0.09 | | |
| SUCRA(aspirin) | 0.78 | | | 0.76 | | | 0.53 | | |
| Average rank (placebo) | 4.13 | | | 4.05 | | | 4.49 | | |
| Average rank (sulodexide) | 3.46 | | | 3.35 | | | 2.73 | | |
| Average rank (DOAC) | 2.05 | | | 2.11 | | | 2.19 | | |
| Average rank (VKA) | 3.49 | | | 3.52 | | | 3.5 | | |
| Average rank (aspirin) | 1.86 | | | 1.96 | | | 2.08 | | |

Abbreviations: DIC, deviance information criterion;DOAC, direct-acting oral anticoagulant; LB, lower bound; OR, odds ratio; P, probability;SUCRA, surface under the cumulative ranking curve; UB, upper bound; VKA, vitamin K antagonist.

**Table 13** Drug-specific results for death from bleeding from the hierarchical model

| Death from bleeding—drug-specific results from hierarchical model—all studies | | | | | | |
|---|---|---|---|---|---|---|
| **Drug** | **Estimate** | **LB** | **UB** | **P(best)** | **SUCRA** | **Average rank** |
| Placebo | 1 | | | 0 | 0.12 | 12.49 |
| Sulodexide | 0.1 | 0 | 7.73 | 0.3 | 0.56 | 6.77 |
| DOAC—Apixaban—2.5mg | 0.13 | 0.01 | 1.45 | 0.05 | 0.67 | 5.29 |
| DOAC—Apixaban—5mg | 0.13 | 0.01 | 1.71 | 0.06 | 0.67 | 5.28 |
| DOAC—Dabigatran | 0.13 | 0.01 | 1.72 | 0.06 | 0.67 | 5.27 |
| DOAC—Edoxaban | 0.12 | 0.01 | 1.27 | 0.07 | 0.7 | 4.85 |
| DOAC—Rivaroxaban—10mg | 0.15 | 0.01 | 1.51 | 0.02 | 0.63 | 5.82 |
| DOAC—Rivaroxaban—20mg | 0.11 | 0.01 | 1.18 | 0.1 | 0.73 | 4.52 |
| VKA—Acenocoumarol | 0.35 | 0.02 | 4.01 | 0.01 | 0.3 | 10.04 |
| VKA—Enoxaparin | 0.33 | 0.02 | 3.57 | 0.01 | 0.34 | 9.59 |
| VKA—Enoxaparin plus warfarin | 0.31 | 0.02 | 2.75 | 0 | 0.35 | 9.50 |
| VKA—Warfarin | 0.42 | 0.03 | 4.33 | 0 | 0.22 | 11.10 |
| VKA—“VKA regimen” | 0.31 | 0.02 | 2.95 | 0.01 | 0.36 | 9.31 |
| Aspirin | 0.1 | 0 | 1.08 | 0.32 | 0.68 | 5.16 |

Abbreviations: DOAC, direct-acting oral anticoagulant; LB, lower bound; P, probability;SUCRA, surface under the cumulative ranking curve; UB, upper bound; VKA, vitamin K antagonist.

**Table 14** Drug-specific results for death by any cause (unspecified) from the hierarchical model

| Death from any cause—drug-specific results from hierarchical model—all studies | | | | | | |
|---|---|---|---|---|---|---|
| **Drug** | **Estimate** | **LB** | **UB** | **P(best)** | **SUCRA** | **Average rank** |
| Placebo | 1 | | | 0 | 0.17 | 11.77 |
| Sulodexide | 0.34 | 0.04 | 1.84 | 0.53 | 0.79 | 3.79 |
| DOAC—Apixaban—2.5mg | 0.55 | 0.25 | 1.07 | 0.03 | 0.66 | 5.41 |
| DOAC—Apixaban—5mg | 0.48 | 0.2 | 0.99 | 0.11 | 0.76 | 4.15 |
| DOAC—Dabigatran | 0.54 | 0.24 | 1.1 | 0.05 | 0.68 | 5.20 |
| DOAC—Edoxaban | 0.54 | 0.18 | 1.29 | 0.09 | 0.66 | 5.44 |
| DOAC—Rivaroxaban—10mg | 0.66 | 0.33 | 1.28 | 0 | 0.51 | 7.39 |
| DOAC—Rivaroxaban—20mg | 0.47 | 0.17 | 1.07 | 0.14 | 0.76 | 4.09 |
| VKA—Acenocoumarol | 0.85 | 0.38 | 2.32 | 0.01 | 0.29 | 10.18 |
| VKA—Enoxaparin | 0.72 | 0.32 | 1.72 | 0.02 | 0.44 | 8.33 |
| VKA—Enoxaparin plus warfarin | 0.75 | 0.37 | 1.47 | 0 | 0.4 | 8.81 |
| VKA—Warfarin | 0.75 | 0.38 | 1.37 | 0.01 | 0.41 | 8.63 |
| VKA—“VKA regimen” | 0.94 | 0.49 | 1.84 | 0 | 0.23 | 10.96 |
| Aspirin | 0.95 | 0.43 | 2.06 | 0 | 0.24 | 10.85 |

Abbreviations: DOAC, direct-acting oral anticoagulant; LB, lower bound; P, probability;SUCRA, surface under the cumulative ranking curve; UB, upper bound; VKA, vitamin K antagonist.

# Supplementary Appendix 5

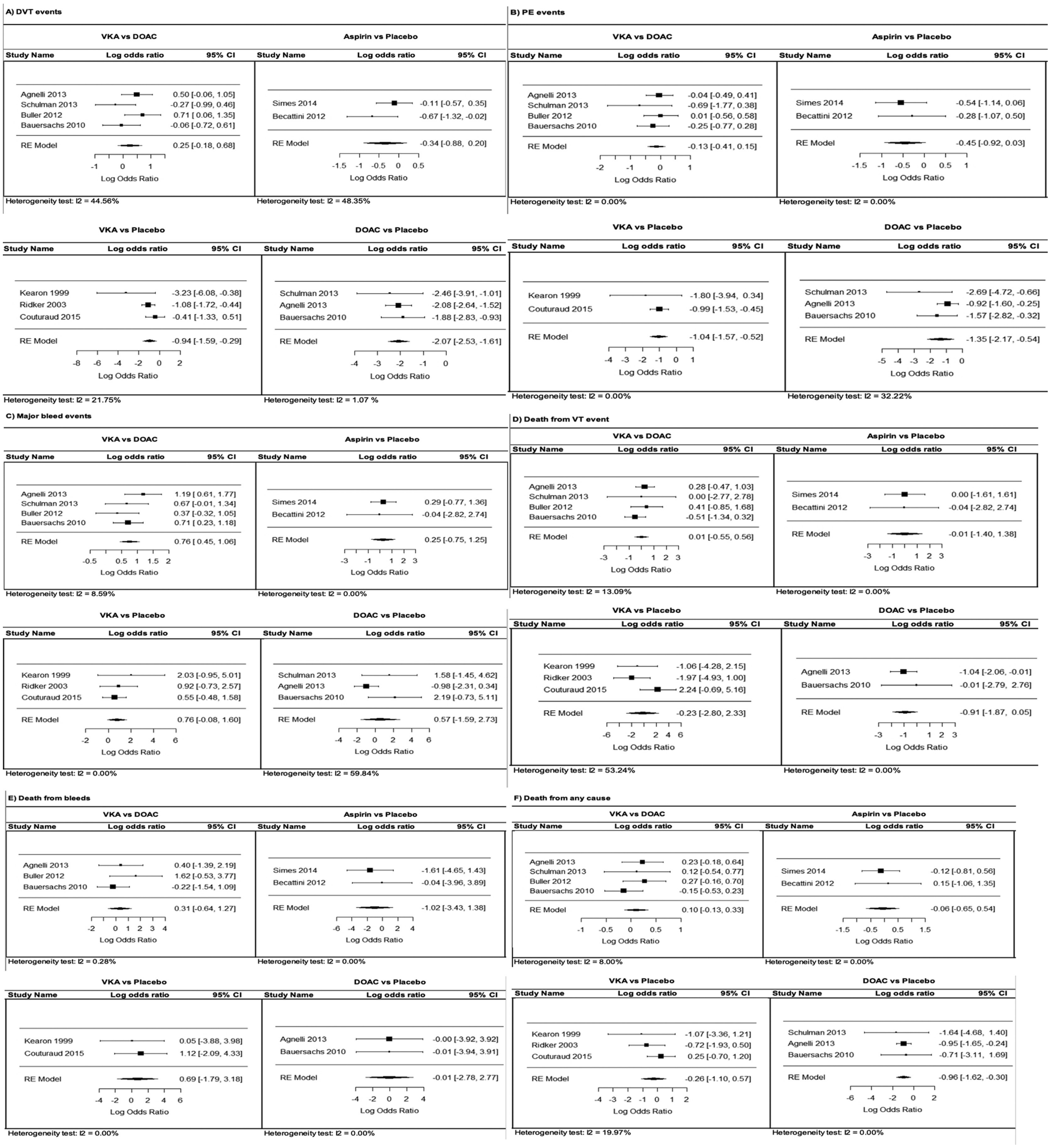

Fig. 1 Forest plots showing the results of pairwise random effects meta-analyses to explore heterogeneity in the comparative studies.

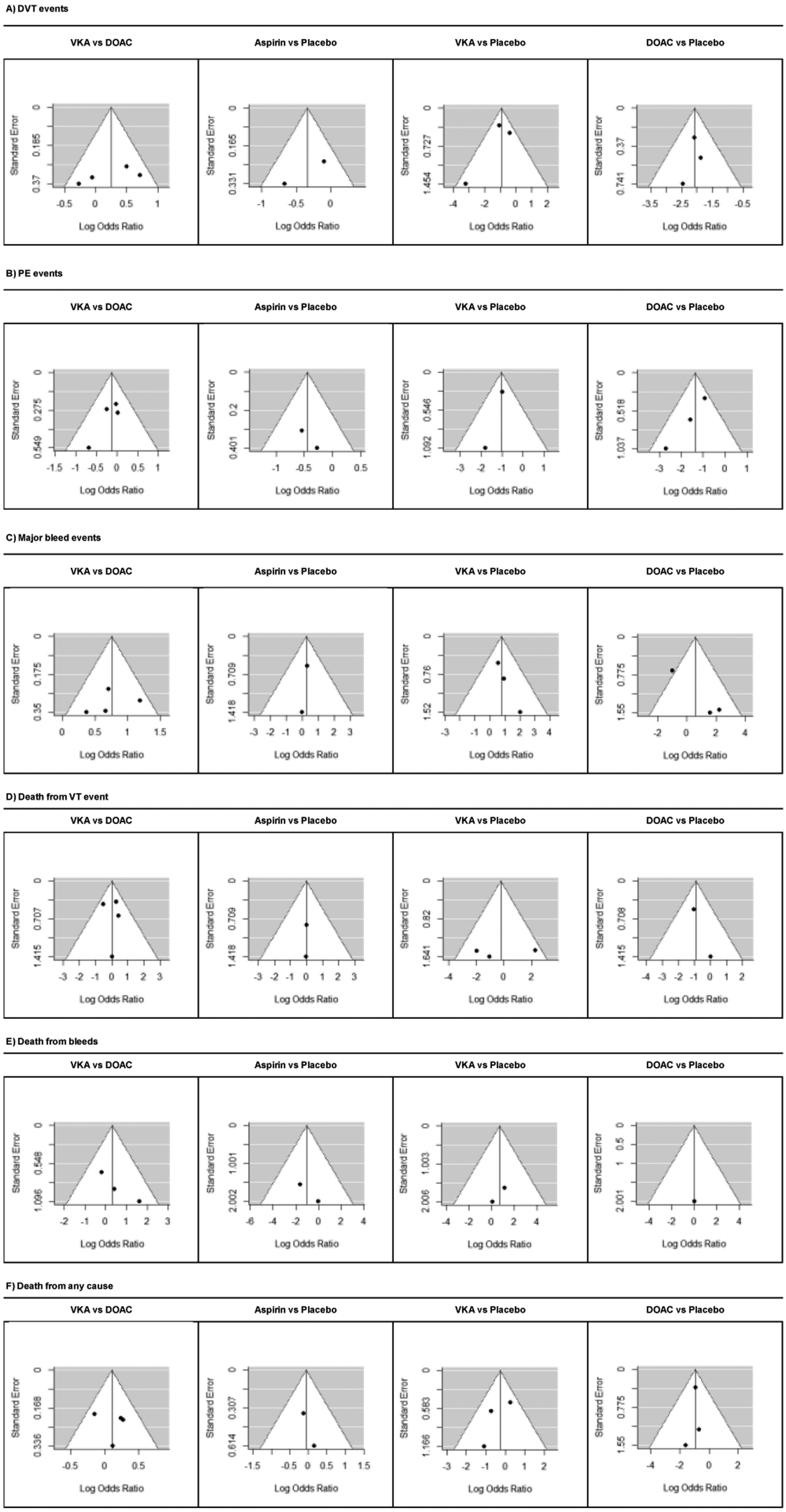


**Fig. 2** Funnel plots showing the results of pairwise random effects meta-analyses to explore publication bias.

| | Random sequence generation (selection bias) | Allocation concealment (selection bias) | Groups similar at the outset (selection bias) | Blinding of participants and personnel (performance bias) | Unexpected imbalances in dropouts (attrition bias) | Selective reporting (reporting bias) | Did the analysis include an intention-to-treat analysis? |
|---|---|---|---|---|---|---|---|
| AMPLIFY [2,14] | + | + | + | + | + | + | + |
| SURVET [6] | + | + | + | + | + | + | + |
| ASPIRE [9,18] | + | + | + | + | + | + | + |
| WARFASA [8,18] | + | + | + | + | + | + | + |
| WODIT-PE [4] | + | – | + | – | + | + | + |
| WODIT-DVT [5] | + | – | + | – | + | + | + |
| Kearon et al, 1999 [13] | + | + | + | + | + | + | ? |
| PREVENT [15] | + | + | + | + | + | + | + |
| RE-MEDY [17] | + | + | + | + | + | + | + |
| RE-SONATE [17] | + | + | + | + | + | + | + |
| PADIS-PE [12] | + | + | + | + | + | + | + |
| AMPLIFY-EXT [2] | + | ? | + | + | + | + | + |
| EINSTEIN-PE [11] | + | – | + | – | + | + | + |
| EINSTEIN-DVT [7] | + | – | + | – | + | + | + |
| EINSTEIN-EXT | + | – | + | – | + | + | + |

**Fig. 3** Summary of quality assessment of the RCTs.RCT, randomized controlled trial.

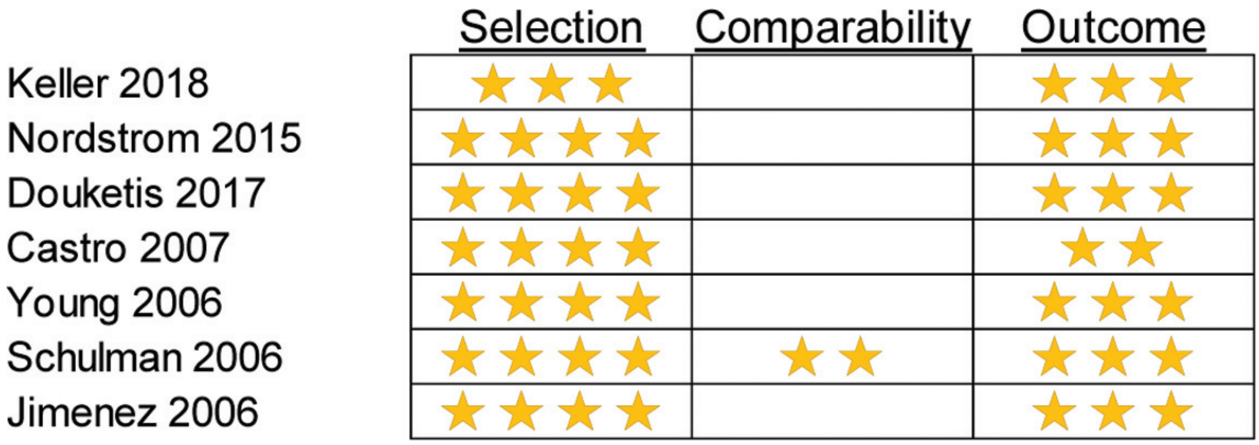

| | Selection | Comparability | Outcome |
|---|---|---|---|
| Keller 2018 | ★★★ | | ★★★ |
| Nordstrom 2015 | ★★★★ | | ★★★ |
| Douketis 2017 | ★★★★ | | ★★★ |
| Castro 2007 | ★★★★ | | ★★ |
| Young 2006 | ★★★★ | | ★★★ |
| Schulman 2006 | ★★★★ | ★★ | ★★★ |
| Jimenez 2006 | ★★★★ | | ★★★ |

**Fig. 4** Summary of quality assessment of the observational studies.
